# A CAM bioimaging model reveals the connection between VEGFA vascular remodeling and enhanced sarcoma progression via tumor secretome

**DOI:** 10.1038/s41598-026-42154-2

**Published:** 2026-03-07

**Authors:** Yuzhe Wang, Wenyu Xue, Nawar Sakr, Averyan V. Pushkarev, Elizaveta N. Mochalova, Margarita Pustovalova, Denis V. Kuzmin, Sergey Leonov

**Affiliations:** 1 Institute of Future Biophysics, Moscow Center for Advanced Studies, Moscow, 123592 Russia; 2 Phystech School of Biological and Medical Physics, Moscow Institute of Physics and Technology, Dolgoprudny, 141701 Russia; 3 Institute of Cell Biophysics of Russian Academy of Sciences, Pushchino, 142290 Russia; 4 Federal Research Center for Innovator and Emerging Biomedical and Pharmaceutical Technologies, Moscow, 125315 Russia

**Keywords:** Sarcoma, Chick embryo chorioallantoic membrane (CAM) model, Tumor cell-conditioned medium (TCM), Bio-imaging, Angiogenesis, VEGFA, Cancer, Cell biology

## Abstract

We developed a sensitive bioimaging system for sarcoma by creating a cell line that stably expresses both Katushka2S fluorophore and NanoLuc luciferase, enabling robust dual tracking of tumor growth and metastasis in the chick chorioallantoic membrane (CAM) model. NanoLuc luciferase was notably more effective than Katushka2S for identifying tumor cell metastases in embryo tissues. Pretreating CAM with tumor cell-conditioned medium (TCM) significantly increased neovascularization, Ki67 expression, tumor volumes, and metastasis of the most difficult-to-establish low-tumorigenic and low-metastatic U2OS cells, indicating that tumor cell secretome actively alter the CAM vascular environment to aid tumor progression. The bead-based multiplex profiling of the TCM demonstrated a notable increase in pro-angiogenic factors. Neutralizing VEGFA, the most abundant factor in the TCM, effectively counteracted vascular and pro-metastatic effects of TCM. In contrast, elevating VEGFA levels brought back the pro-tumorigenic effects of TCM. This study reveals the importance of tumor cell secretomes in creating the vascular niche in CAM and points to VEGFA as a target to prevent secretome-induced angiogenesis and sarcoma development. Furthermore, our optimized CAM model permits continuous tumor growth and metastasis monitoring in embryonic development, providing a reliable platform for prognostic studies of sarcoma treatments in ongoing anti-angiogenic and multikinase inhibitors trials.

**Supplementary Information:**

The online version contains supplementary material available at 10.1038/s41598-026-42154-2.

## Introduction

Sarcomas comprise a diverse group of tumors derived from mesenchymal tissues, with around 80% originating in soft tissues and the remaining 20% arising from bone^1^. Studies have shown that the relative 5-year survival rate for osteosarcoma (OS) patients is approximately 50% ^2,3^. Fibrosarcoma (FS) is a rare yet aggressive tumor originating from mesenchymal cells, particularly fibroblasts found in connective tissue. Fibrosarcoma has a poor prognosis due to its aggressive behavior, resistance to radiation and chemotherapy, and high recurrence rates^4,5^. These features underscore the urgent need to elucidate the molecular mechanisms underlying sarcoma progression and to identify therapeutic vulnerabilities.

The tumor microenvironment, made up of blood vessels, supporting tissues, and substances from tumors, is essential for tumor growth and spread. Therefore, realistic models that mimic these interactions are vital for studying the mechanisms involved and evaluating therapies. The chick embryo chorioallantoic membrane (CAM) model provides a highly vascularized, immunodeficient, and easily observable system for studying tumor-vascular interactions efficiently and affordably^6–9^.

Although the CAM model has been extensively applied to assess tumor growth and angiogenesis^7,10^, it has been rarely utilized to elucidate the molecular mechanisms underlying the pre-engraftment remodeling of the host vascular niche by tumor cells. Emerging evidence suggests that tumor cell–derived secretome—comprising cytokines, growth factors, and extracellular vesicles—play an active role in preconditioning the microenvironment before tumor cell colonization^11,12^. Among these components, vascular endothelial growth factor A (VEGFA) is a key regulator of angiogenesis and vascular permeability. However, its specific contribution to secretome-mediated vascular remodeling during the early stages of sarcoma implantation has not been fully elucidated.

In this study, we developed a dual-reporter bioimaging system to monitor sarcoma progression in the CAM model and studied the effect of tumor cell–conditioned medium (TCM) on tumor growth and spread. We demonstrate that TCM enhances neovascularization, vascular leakage, and metastatic spread of low-tumorigenic, low-metastatic U2OS cells. Multiplex profiling found VEGFA to be the most abundant pro-angiogenic factor in TCM. Functional tests showed that blocking VEGFA reversed the vascular and pro-metastatic effects caused by TCM. These findings show a connection between the sarcoma secretome and early vascular niche remodeling, suggesting that VEGFA could be a potential target for interrupting tumor growth driven by the secretome.

## Results

### Optimization of CAM-Based multimodal bioimaging for tumor growth and metastasis quantification

A robust and quantitative bioimaging strategy is essential for monitoring tumor growth and metastasis in the CAM model. Therefore, we systematically evaluated fluorescence- and luminescence-based imaging modalities to optimize signal detection and quantification.

**Fig. 1:**
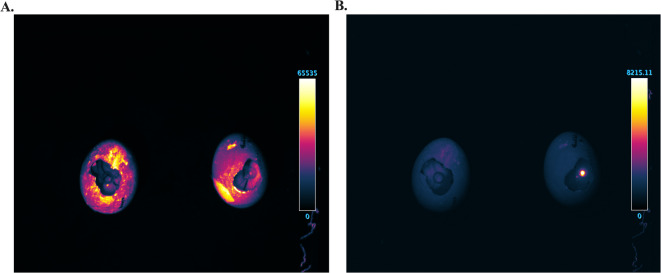
Comparison of GFP and Katushka2S fluorescence. Imaging of tumor cell nodules on CAM formed at EMD 16 after implantation of 1.5 × 10^6^ of WEHI-164-Kat2S-T2A-Nluc-GFP cells. Fluorescent signals of GFP (**A**) and Katushka2S (**B**) (control (sham operated eggs) on the left, experimental (with implanted tumor cells) group on the right) were obtained using LumoTrace^®^ Fluo (Abisense LLC, Russia) imaging system.

Imaging of tumor nodules formed by WEHI-164-Kat2S-T2A-Nluc-GFP cells at EMD16 revealed pronounced background autofluorescence in the GFP channel, which hindered reliable identification of tumor nodules on the CAM surface. In contrast, Katushka2S fluorescence displayed a markedly improved signal-to-noise ratio, allowing clear delineation of tumor regions against the CAM background (Fig. 1).

**Fig. 2:**
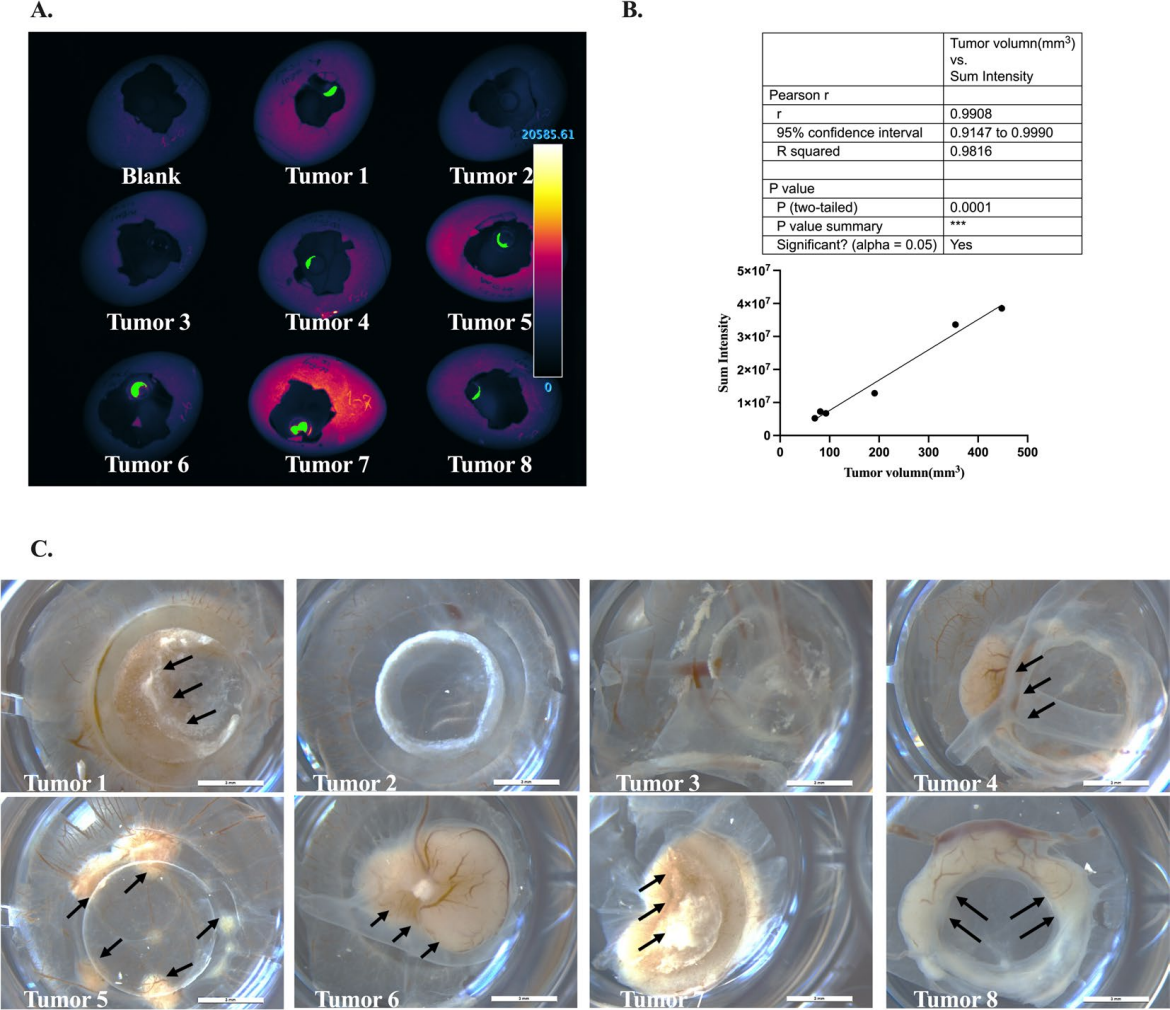
In ovo tumor formation and quantification at EMD 16 following implantation of 1.5 × 10⁶ WEHI 164-Kat2S-T2A-Nluc cells. (**A**) Fluorescence imaging of tumor-bearing CAMs by LumoTrace^®^ Fluo (Abisense LLC, Russia). Green regions represent tumor ROIs identified by Icy software, and color scale indicates fluorescence intensity. (**B**) Correlation between integrated fluorescence intensity (Sum Intensity) within tumor ROI and tumor volume. (**C**) Bright-field images of tumor nodules. Visible tumors are indicated by black arrows. Scale bars = 3 mm.

Quantitative validation of Katushka2S-based measurements was achieved by defining tumor regions of interest (ROIs) on the CAM surface and calculating the integrated fluorescence intensity. Comparison with ex vivo tumor measurements demonstrated a strong positive correlation between ROI fluorescence intensity and tumor nodule volume (Pearson’s R² = 0.9984, *p* < 0.000001), confirming that the measured signal reflects tumor burden rather than nonspecific light reflection (Fig. 2; Supplementary Fig. 3).

**Fig. 3:**
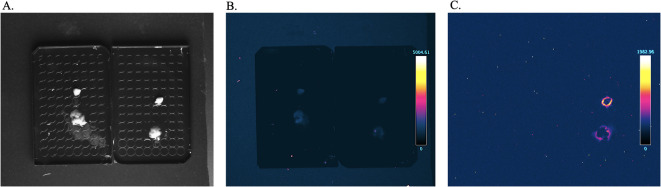
Imaging of tumor cell metastases in chicken embryonic organ tissues formed at EMD 16 after implantation of 3 × 10^6^ U2OS-Kat2S-T2A-Nluc cells. Bright-field (**A**), Katushka2S fluorescent (**B**) and NanoLuc luminescent (**C**) images of embryonic tissues (left column - control group, right column - experimental group: upper panel - heart, lower panel - liver).

For the assessment of distant metastases, NanoLuc luciferase–based bioluminescence imaging was additionally employed as a highly sensitive detection modality. Katushka2S fluorescence enabled reliable longitudinal visualization of primary tumor growth and metastatic dissemination in the CAM model, whereas NanoLuc-based imaging offered enhanced sensitivity for end-point detection of low-burden metastatic lesions in embryonic organs, including the heart and liver (Fig. 3B, C). Together, these observations support a complementary multimodal imaging strategy, in which fluorescence and luminescence approaches provide synergistic information within the CAM model.

## TCM pretreatment facilitates tumor take rates and metastases in spontaneous metastasis CAM model

**Fig. 4:**
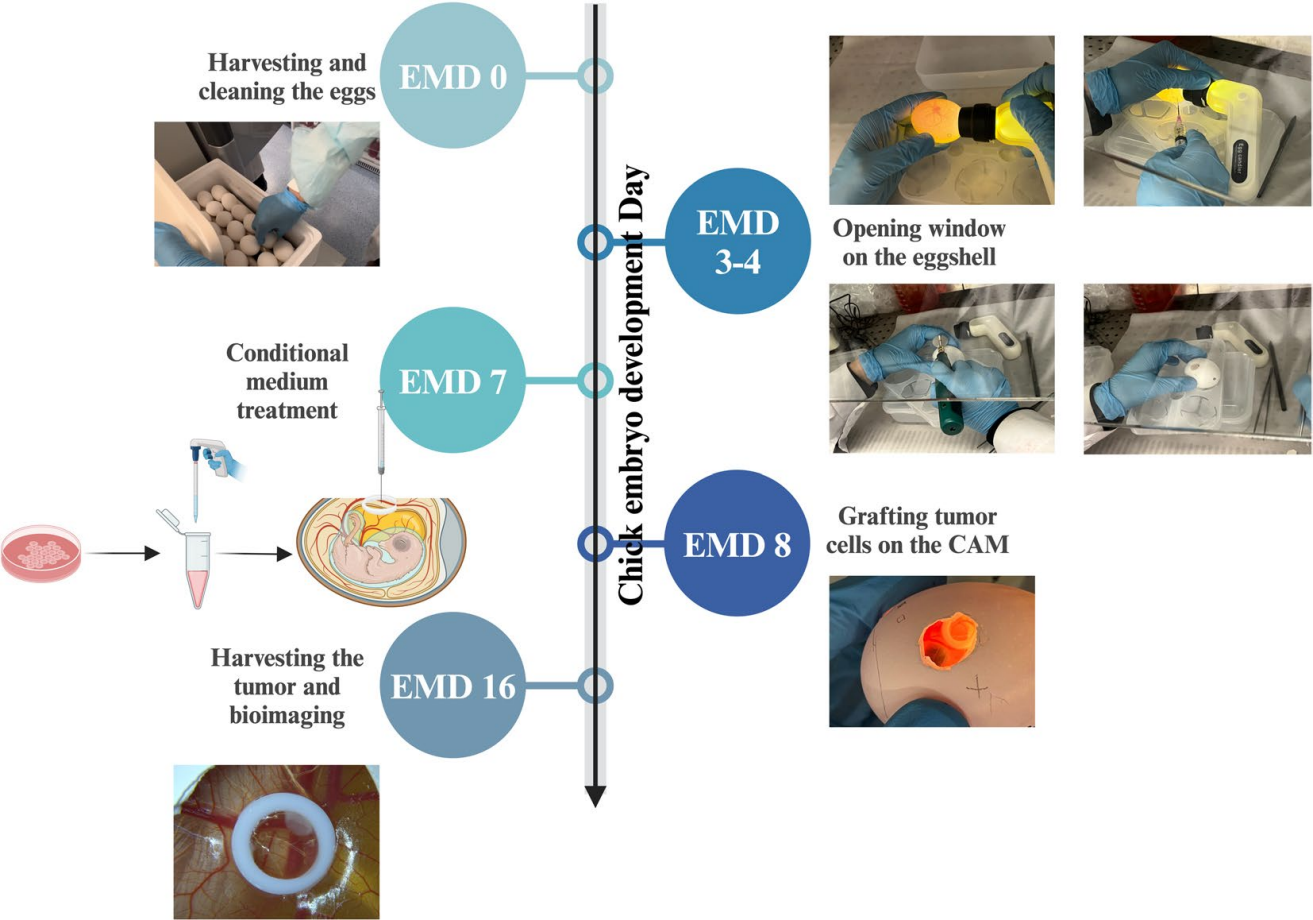
Overview of the TCM-pre-treated CAM (TCM-CAM) assay schedule from EMD 0 to EMD 16.

**Fig. 5:**
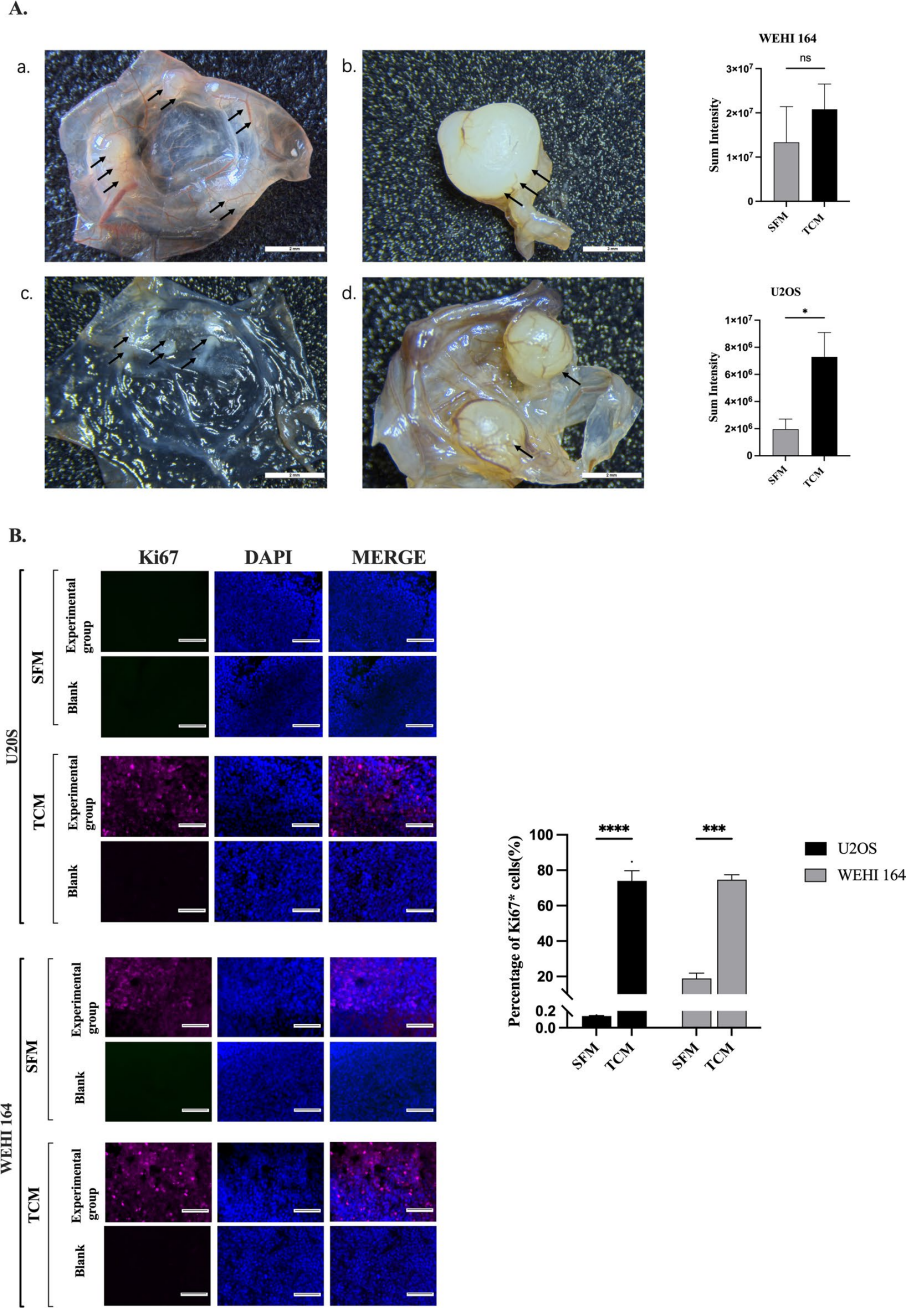
TCM pretreatment promotes tumor nodule formation and proliferation in the CAM model. (**A**) Representative macroscopic images of in ovo tumor nodules (black arrows) formed by WEHI-164-Kat2S-T2A-Nluc-GFP cells (a, b) and U2OS-Kat2S-T2A-Nluc-GFP cells (c, d) at EMD16 after implantation on the CAM. CAMs were pretreated with tumor-conditioned medium (TCM; b, d) or serum-free medium (SFM-treated group; a, c) prior to tumor cell implantation. Quantification of Katushka2S-T2A fluorescence intensity, reflecting tumor burden, is shown in the right-hand panels. Scale bars = 3 mm. (**B**) Representative immunofluorescence staining of Ki67 (pink) and nuclei(DAPI, blue) in tumor nodules derived from SFM-treated and TCM-pretreated CAMs for U2OS and WEHI-164 cells. Quantification of Ki67-positive cells is shown on the right. Scale bar = 75 μm. Error barsrepresent standard errors of the mean. Two-sided p-value for Student’s t-test and 2-way ANOVA: * < 0.05 *** < 0.001; **** ≤ 0.0001

An overview of the TCM-pretreated CAM experimental workflow is provided in Fig. 4 to outline the timing of CAM manipulation, TCM treatment, and tumor cell implantation. We hypothesized that pretreatment of the CAM with TCM could facilitate tumor progression on the CAM surface and promote metastasis of tumor cells within the chicken embryo. The results demonstrated that CAMs treated with TCM exhibited enhanced tumor take rate compared to the SFM-treated group. Additionally, tumors in the TCM-treated group were more solid and homogeneous, akin to forming regular tumor spheres (Fig. 5A-b, d).

An increase in Ki67 expression was observed following TCM treatment (Fig. 5B), indicating that more cells were actively undergoing cell division. This suggests that TCM may contain factors that promote the proliferative activity of both tumor cells, thereby facilitating their tumor take rates in ovo.

**Fig. 6:**
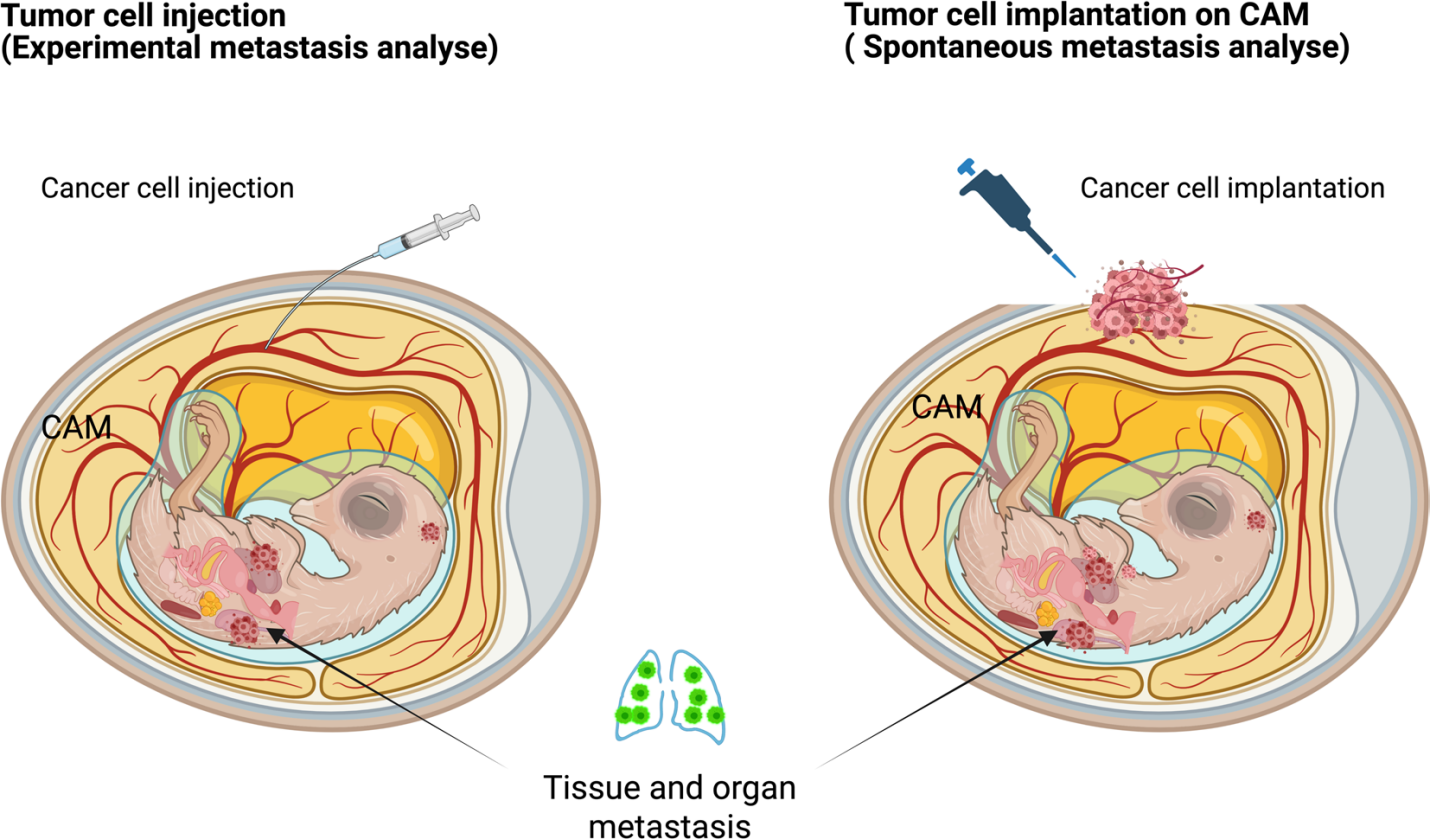
Tumor metastasis models in chicken embryos. Left – the experimental metastasis model; Right – the spontaneous metastasis model^44^.

**Fig. 7:**
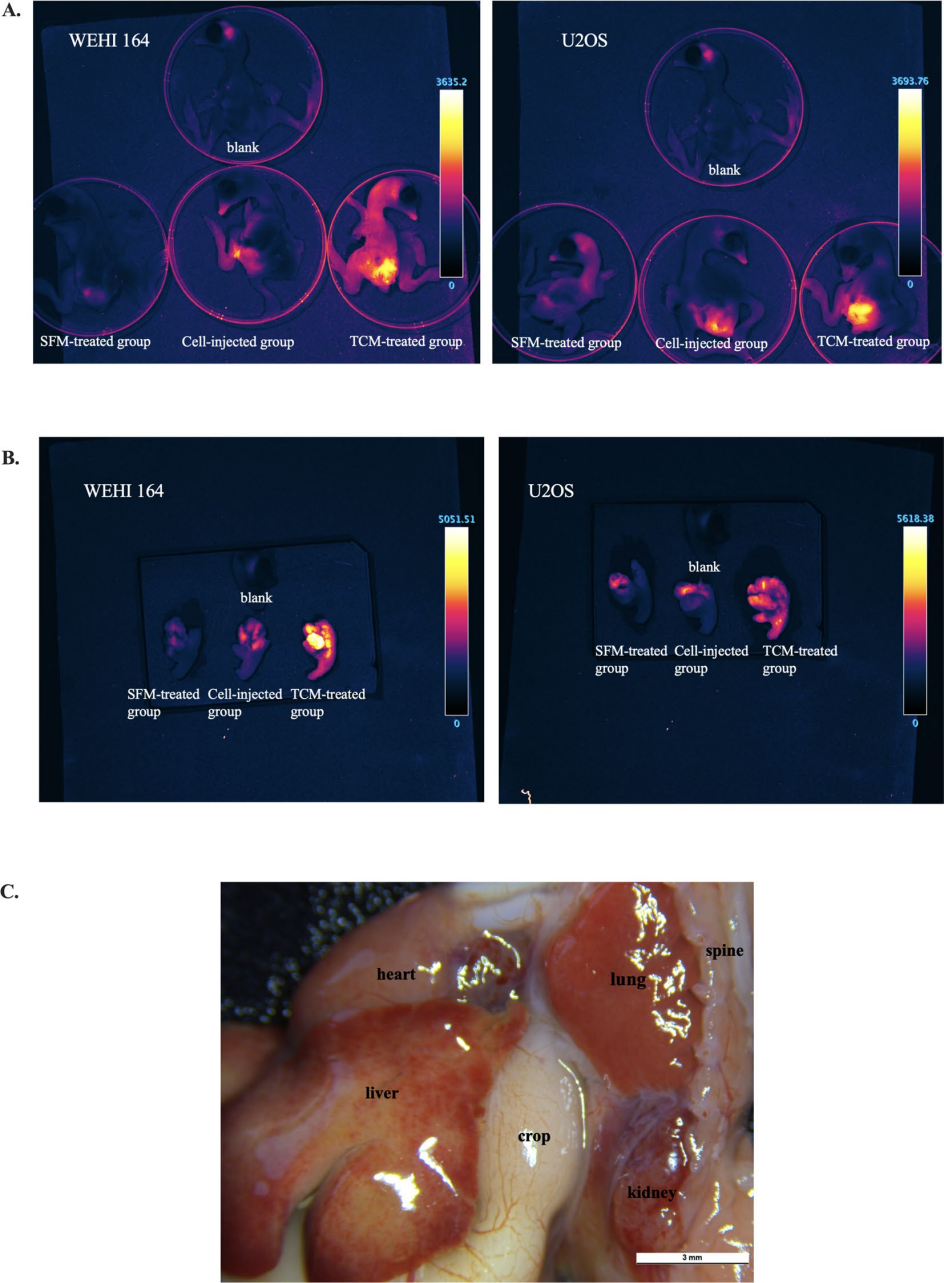
Bioimaging of spontaneous and experimental metastasis at EMD16 after implantation of reporter sarcoma cell lines. CAMs were subjected to one of three conditions: (i) Control spontaneous metastasis group, in which CAMs were pretreated with serum-free unconditioned medium(SFM); (ii) TCM-treated spontaneous metastasis group, in which CAMs were pretreated with tumor-conditioned medium (TCM); (iii) Cell-injected group (positive control), in which tumor cells were intravenously injected into the chorioallantoic vein following a previously established protocol^58^, without TCM pretreatment. Metastatic signals were recorded in the Katushka2S fluorescence channel. (**A**) Whole-embryo imaging. (**B**) Imaging of isolated embryonic organs (macroscopic views shown in panel (**C**). Scale bars = 3 mm.

Metastasis of OS cells in chick embryonic organs was compared under three paradigms, schematically illustrated in Fig. 6: (1) an SFM-treated spontaneous metastasis group, in which CAMs were pretreated with serum-free medium, (2) experimental metastasis model (Cell-injected group), and (3) TCM-treated spontaneous metastasis model (TCM-treated group). TCM treatment promotes the metastasis of tumor cells from the CAM surface to the organs of chick embryos (Fig. 7). We found that tumor cell metastasis first occurred in the heart and liver tissues, followed by the lungs and kidneys. This sequence of metastasis may be linked to the developmental timeline of organ formation in chick embryos.

## TCM pretreatment enhances angiogenesis and vascular permeability in the spontaneous metastasis CAM model

To explore the mechanisms by which TCM promotes tumorigenesis and metastasis on the CAM, we evaluated the effect of TCM pre-treatment on angiogenesis and vascular permeability within the CAM microenvironment.

**Fig. 8:**
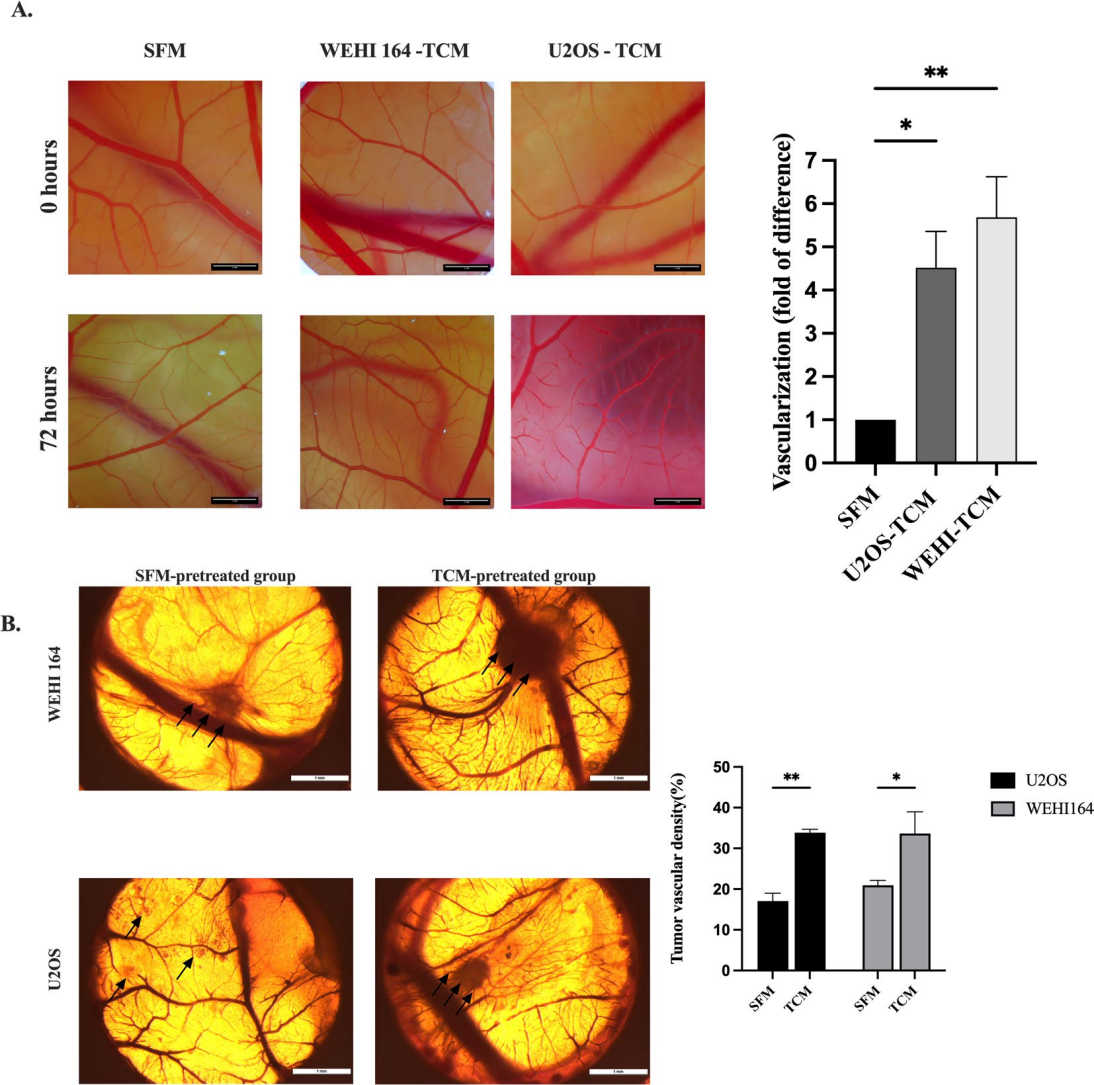
TCM pretreatment induces vascular remodeling and enhances tumor-associated vascularization in the CAM model. (**A**) Representative CAM images captured at 0 and 72 h after pretreatment with serum-free medium (SFM-treated group) or tumor-conditioned medium (TCM) derived from WEHI-164 or U2OS cells. Quantification of CAM vascularization, expressed as fold change relative to the SFM-treated group, is shown in the right panel. (**B**) Representative ex ovo CAM images of tumor nodules at EMD12 formed by WEHI-164 or U2OS cells on CAMs pretreated with SFM or TCM. Quantification of tumor vascular density (%) on the right panel. Arrows indicate tumor nodule locations. Scale bars = 1 mm. Two-sided p-value for 1-way ANOVA and 2-way ANOVA: * < 0.05 ** < 0.01; *** < 0.001.

Over a 72-hour period, angiogenesis was compared between CAMs pre-treated with serum-free medium (SFM) and tumor-conditioned medium (TCM). We found that minimal scratching followed by serum-free medium treatment did not induce significant micro-vessel formation in the CAM microenvironment when compared to the untreated control (supplementary Fig. 4). In contrast, the TCM-pretreated group, after minor scratching, exhibited a notably denser microvascular network in the CAM as indicated by 4,5- and 5,5-fold increase in vascularization induced by TCM from both U2OS and WEHI-164 cells, respectively (Fig. 8A). This indicate that sarcoma cells, when cultured in serum-free medium, may secrete pro-angiogenic factors into the extracellular milieu.

Further validation of the above hypothesis was carried out by collecting tumors on EMD12 and performing angiography. The results showed that, compared to the medium pre-treatment group, TCM pre-treatment led to the formation of larger solid tumors and denser tumor vasculature (Fig. 8B). This finding highlights that a rich vascular microenvironment in the early stages of tumor formation creates favorable conditions for rapid tumor proliferation. Hence, TCM pre-treatment significantly enhances tumor formation in the spontaneous metastasis CAM model, even with cell lines that typically struggle to develop tumors in vivo, thanks to its powerful impact on neovascularization.

**Fig. 9:**
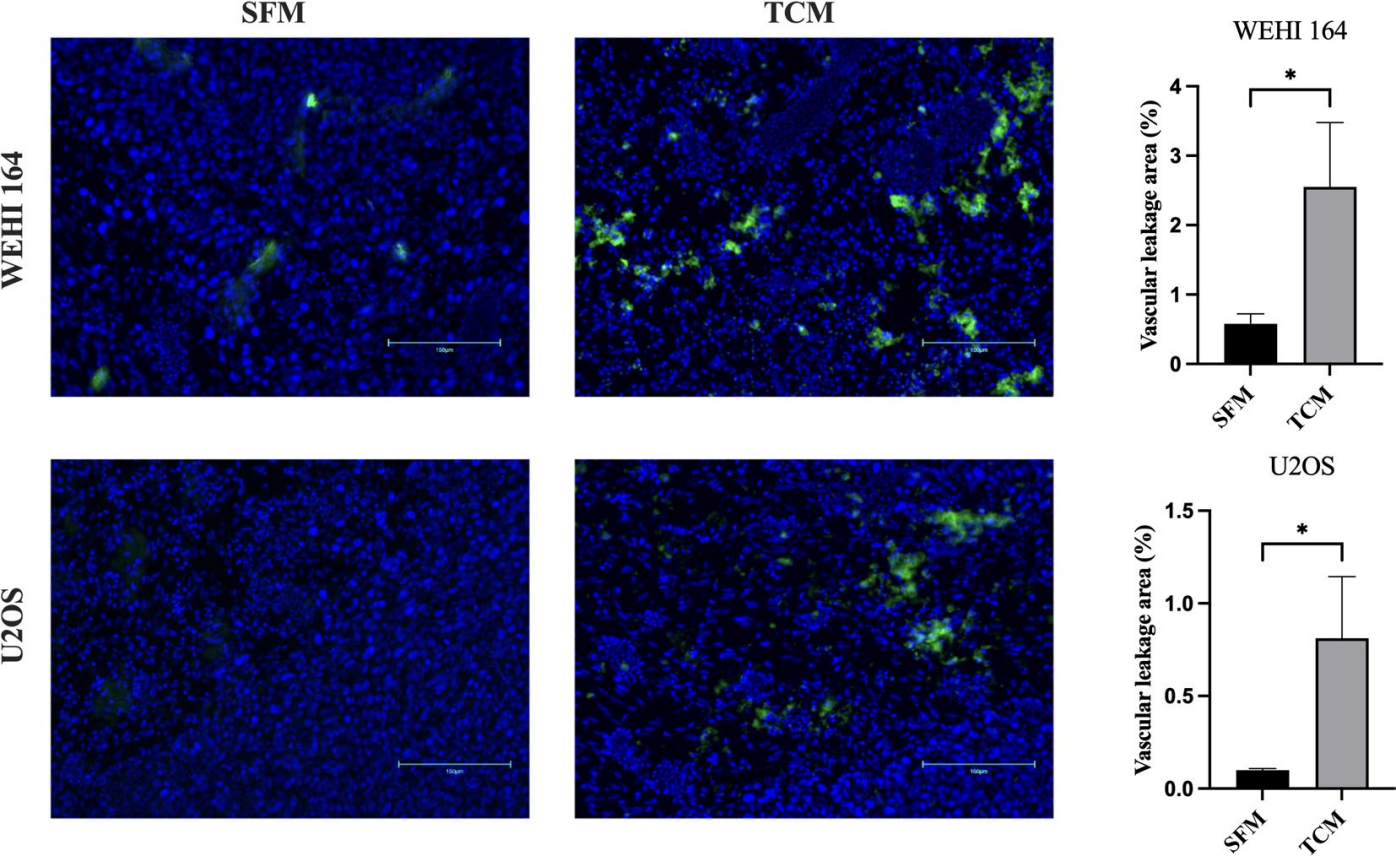
Assessment of tumor-associated vascular permeability using a FITC-dextran leakage assay in the CAM model. Representative fluorescence images of CAM tumor tissues from WEHI-164 (top row) and U2OS (bottom row) groups following pretreatment with serum-free medium (SFM) or tumor-conditioned medium (TCM). FITC-conjugated dextran (70 kDa) leakage into the tumor tissue is shown in green, and nuclei are counterstained with DAPI (blue). Quantification of vascular leakage area (%) is shown in the right panels. Scale bars = 150 μm. Error bars represent standard errors of the mean. Two-sided p-value for Student’s t-test: * < 0.05.

To assess tumor-associated vascular permeability, a FITC–dextran leakage assay was performed on day 8 after tumor cell implantation (EMD16). In both the U2OS and WEHI-164 models, TCM pretreatment resulted in a broader distribution of FITC fluorescence within tumor tissues, indicating increased vascular leakage compared with control conditions (Fig. 9).

**Targeting VEGF of the TCM inhibits neovascularization**,** vascular permeability**,** growth and metastasis of human OS cells in vivo.**

Key components of the tumor cell secretome include cytokines, chemokines, growth factors, and other soluble mediators^13^. Among these, VEGF is a central pro-angiogenic factor that promotes neovascularization, modulates immune responses, and supports tumor progression. To comprehensively characterize secretome constituents, we performed a systematic analysis of 41 cytokine and chemokine biomarkers in both control medium and tumor-conditioned medium (TCM) derived from each individual cell line. Quantification of these secreted factors provides insight into the biological processes shaping tumor–microenvironment interactions and offers a useful framework for interpreting the CAM microenvironmental changes observed in this study.

**Fig. 10:**
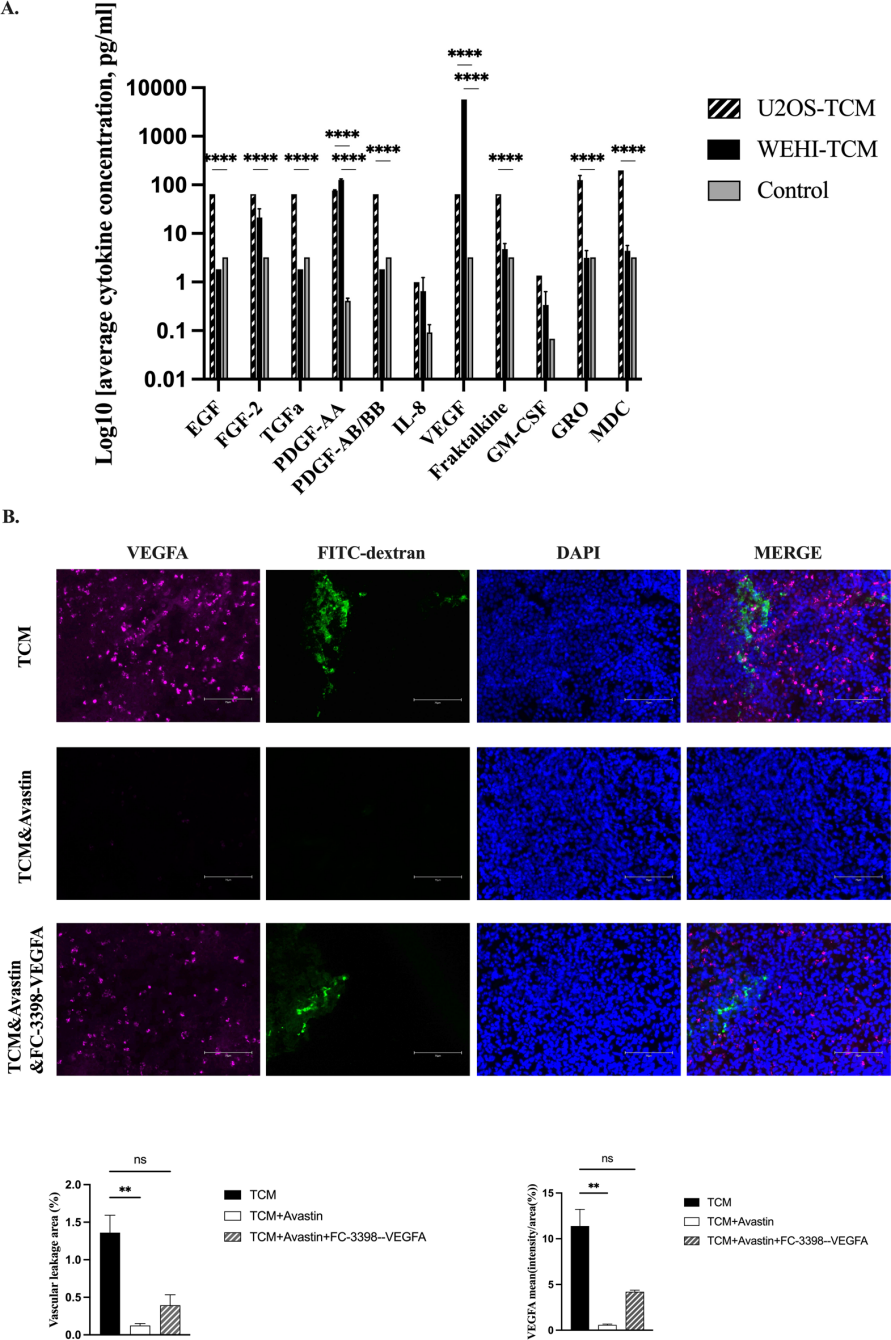
Cytokine profiling of tumor-conditioned media and VEGF-dependent regulation of vascular permeability in the CAM tumor model. TCM cytokine profiling (**A**) and immunofluorescent quantification of vascular leakage and VEGF expression (the bottom panel on **B**) in tumor nodule tissues at EMD 16 after implantation of U2OS reporter cells after CAM treatment with TCM (TCM), TCM neutralized by Avastin (TCM+Avastin) and TCM neutralized by Avastin and VEGF recovered (TCM+Avastin + FC-3398-VEGFA). Scale bars = 75 μm. Two-sided p-value for 1-way and 2-way ANOVA: ns, *P* > 0.05; * < 0.05; *** < 0.001; **** ≤ 0.0001.

Multiplex analysis showed that several angiogenesis-related cytokines in tumor cell secretome (TCM) were significantly elevated compared to the control medium (Fig. 10A).

Interestingly, the only molecules increasingly secreted by both WEHI 164 and U2OS cells were PDGF-AA and VEGF. VEGF is crucial for understanding tumor growth and changes in blood vessel formation. It is particularly noteworthy that the secretome derived from WEHI 164 cells exhibits the highest level of its expression. This indicates that the secretory profile of these cells may be important in shaping the tumor microenvironment in CAM. Consequently, the elevated levels of VEGF could be linked to the enhanced progression of OS tumors. The results indicate a need for more research on VEGF’s role in the TCM secretome, which shows significant therapeutic potential.

We next compared VEGF expression and vascular permeability in tumor nodule tissues following TCM treatment, in the presence or absence of Avastin, a clinically approved VEGF-neutralizing antibody, to evaluate the contribution of VEGF to TCM-induced vascular leakage. Avastin-mediated VEGF neutralization in TCM markedly reduced both VEGF expression and FITC–dextran leakage within tumor tissues after U2OS cell implantation on the CAM (Fig. 10B). Transient restoration of VEGF expression in these cells partially but significantly rescued VEGF levels and increased vascular permeability, confirming the functional role of VEGF in regulating tumor-associated vascular leakage in this in vivo model.

**Fig. 11:**
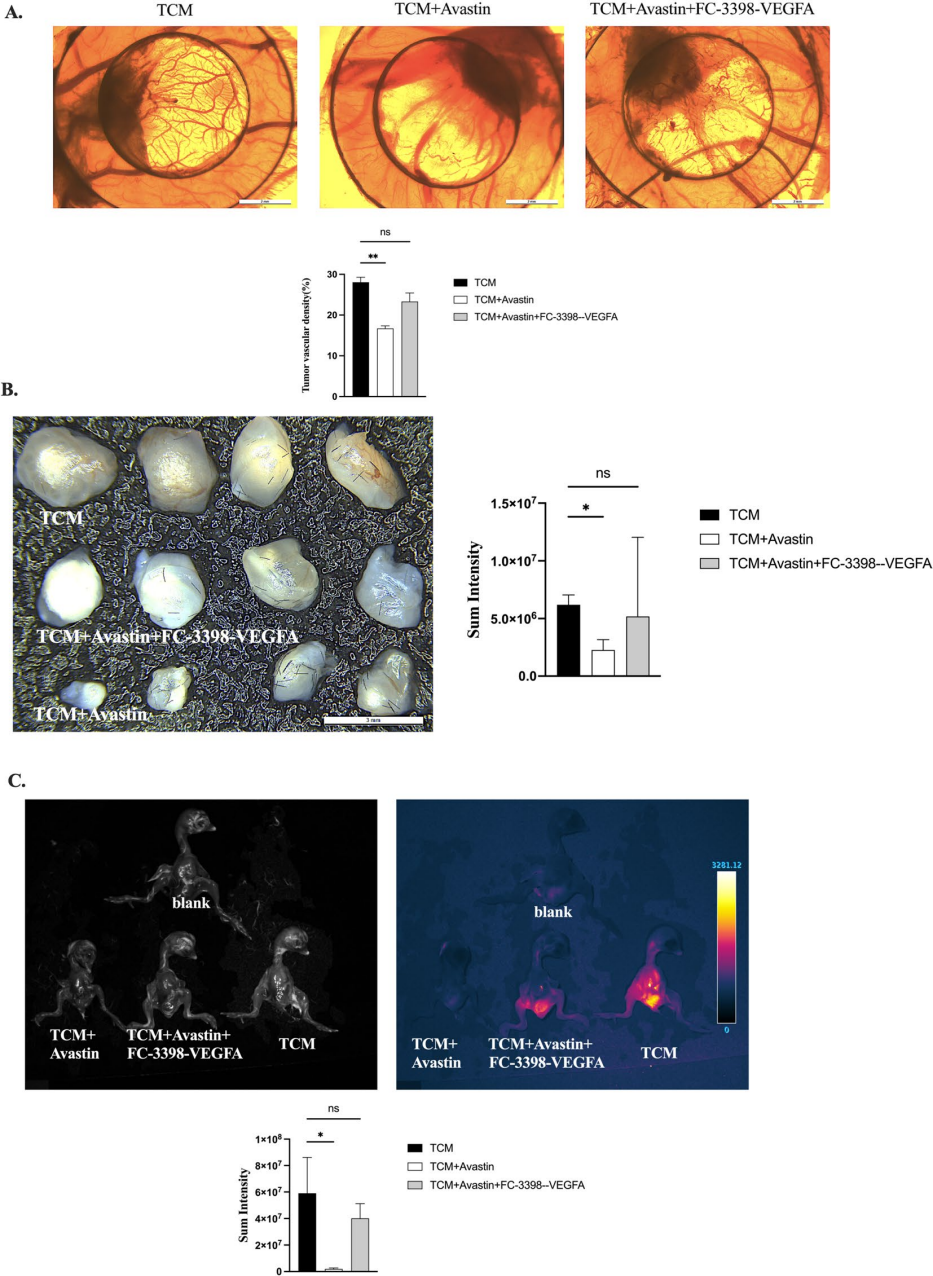
Tumor growth, neovascularization, and metastasis of U2OS reporter cells in the CAM model. (**A**) Representative macroscopic images of CAM tissue showing tumor-associated neovascularization (top panels) and corresponding quantitative analysis of vascular density (bottom graph). Scale bars = 2 mm. (**B**) Representative brightfield images of excised tumor nodules from the indicated treatment groups. Scale bar = 3 mm. (**C**) Representative brightfield images (left) and corresponding Katushka2S-T2A fluorescence images (right) illustrating tumor burden and metastatic dissemination in chicken embryos under the indicated conditions. Quantification of Katushka2S-T2A fluorescence-indicated tumor volume and metastases are presented on the right of B and C panels, respectively. Two-sided p-value for Student’s t-test: * < 0.05; ** < 0.01; NS - not significant.

Based on the above vascular permeability results, we further investigated the effects of VEGF blockade on CAM neovascularization, tumor growth, and metastatic dissemination following TCM treatment. Avastin-mediated VEGF neutralization significantly reduced neovascularization in the CAM tissue surrounding tumor nodules after U2OS cell implantation (Fig. 11A). Consistent with these vascular changes, VEGF neutralization also significantly decreased tumor nodule volumes (Fig. 11B) and effectively abolished metastatic dissemination, as demonstrated by whole-embryo and organ-level bioimaging (Fig. 11C). Conversely, restoration of VEGF expression markedly enhanced CAM neovascularization, which was accompanied by recovery of tumor growth and metastatic spread. The findings emphasize the importance of VEGF in tumor-associated neovascularization, tumor growth and metastasis, confirming it could be a good target for the treatments.

## Discussion and conclusion

In sarcoma clinical research, real-time quantification of tumor growth and metastasis, along with a comprehensive understanding of interactions with the host microenvironment, remains a significant challenge 14–17. While traditional animal models have provided valuable insights into the biological characteristics of sarcomas^18^–^20^, their complex procedures, lengthy experimental timelines, and high costs limit the efficient study of dynamic tumor behaviors 21–23. In this context, CAM models combined with high-sensitivity imaging platforms have introduced new advancements and breakthroughs in different tumor studies 24,25. Few attempts to use the CAM model for studying osteosarcomas have faced challenges, as some cell lines did not produce tumor nodules large enough to be measured accurately. Based on published data, such cell lines include well-known human osteosarcoma lines such as HOS, SaOS and U2OS, known for their lowest tumor volume and take rates even under optimized conditions of the CAM method 6. This makes it difficult or even impossible to obtain reproducible results where quantitative verification of anti-tumor effects is needed using this in vivo model. At the same time, several mouse osteosarcoma cell lines have yet to be evaluated for their suitability in the CAM model.

We have significantly advanced the pioneering work of Olivier De Wever and his team from Ghent University, demonstrating the CAM model’s effectiveness in studying human sarcomas^26^,^27^. In particular, from technical stand-point, we consistently achieved measurable nodule formation with the U2OS cell line, which usually still produced very small tumor nodules despite following the Olivier De Wever group’s optimized conditions^27^. We accomplished this without a No. 11 scalpel 26 or glass cannula 27, which made the process of removing the epithelial cell layer difficult to replicate without injury. We didn’t need to use costly and less consistent methods for immobilizing tumor cells in Matrigel, which is an ECM gel from mouse sarcoma, during implantation. Consequently, we achieved better engraftment of OS cell implants and significantly larger tumor nodules, which helps us measure their sizes and the spreading of metastases in chicken embryos more accurately.

Unlike the studies by Olivier De Wever et al. ^26,27^, which used microscopic histology for tumor analysis, our method emphasizes visual and quantitative evaluation of tumor nodules and metastases in chicken embryos, avoiding time-consuming immunohistochemistry techniques. Moreover, their studies focused on macroscopic scoring of angiogenesis in tumor tissue with a tedious scale: 0 = no changes, 1 + = few vessels converging to the tumor, and 2 + = increased density and length of vessels. This is an operator-dependent approach and does not fully reflect angiogenesis induced by tumor cells and their secretome. Our approach allows for a better quantitative analysis of angiogenesis in the CAM tissue near tumor nodules, influenced by tumor growth and cell secretions.

In the present study, we optimized a bioimaging system using the CAM model for the U2OS human osteosarcoma cell line and the mouse fibrosarcoma cell line WEHI-164, which has not been studied for tumor formation in the CAM model before. For the first time, we created a lentiviral construct, PLKO-Katushka 2 S-T2A-Nluc, to develop cell line that stably expresses a far-red fluorophore and a luminescent reporter, allowing for whole-body and metastasis bioimaging in the CAM model. This imaging system enables precise tracking of tumor volume changes, allowing researchers to monitor primary tumor progression after treatment with different therapies. In addition, the platform facilitates the observation of tumor metastasis from the CAM surface to chick embryonic tissues. Our data indicate NanoLuc^™^ luciferase imaging is more advantageous in detecting trace amounts of tumor metastases. This imaging method has low background noise, allowing for intravenous injection of substrates without harming the animals, enabling in vivo studies of tumor cell metastasis^28^.

To enhance tumor volumes and take rates, we pretreated CAM with tumor cell conditioned medium (TCM) containing tumor cell secretome before injecting suspension of syngeneic tumor cells. This approach has yet to be evaluated within the CAM model. Our study demonstrates for the first time that pre-treatment of CAM with TCM, combined with scratching the CAM membrane surface before cell inoculation, significantly enhances the formation of more regular tumor spheres by both tested sarcoma cell lines. The promotion was especially dramatic for the most difficult to establish U2OS tumors (Fig. 5A). Moreover, Ki67 expression in tumor nodule tissue significantly increased after TCM treatment compared to the untreated control group (Fig. 5B). This new data indicates that more tumor tissue resident cells are actively dividing after TCM treatment. Furthermore, we also demonstrated that CAM pretreatment with TCM significantly improve both whole-body and embryo organ bioimaging of primary tumor metastasis established by both sarcoma cell lines (Fig. 7). These findings led us to explore the factors and mechanisms that caused such a significant change in sarcoma tumor development in CAM.

Angiogenesis involves the breakdown of junctions between endothelial cells, enhancing leakage and causing morphological changes that aid in their migration 29,30. These processes are carried out by proteins released within the tumor microenvironment. Accordingly, we show that pretreating the CAM with TCM, then adding tumor cells in suspension, significantly enhances the growth of a strong blood vessel network (Fig. 8) and boosts the leakage of blood vessels (Fig. 9) in the early stages of tumor growth. This likely meets the fast metabolic needs of growing tumor tissue and encourages solid tumor formation and metastasis on the CAM. In humans, new blood vessels allow tumor cells to enter the bloodstream and spread to distant organs, leading to a correlation between tumor vascularity and poor prognosis 31. Our in ovo experiments demonstrated that TCM pre-treatment of the CAM promotes the formation of a more abundant capillary network within its microenvironment, thereby facilitating sarcoma cells in acquiring sufficient resources during the early stages of tumorigenesis 32. This process enhances tumor growth (Fig. 5A) and metastasis (Fig. 7) in the CAM model. Additionally, our approach reduces the reliance on expensive materials such as matrix gels and simplifies the procedure, offering a novel strategy for CAM model in oncology analysis.

While the CAM model was widely used for investigation of tumor angiogenesis 33,34, the only past research showed that TCM promotes blood vessel growth in a CAM model, albeit without compulsively examining the effect of TCM on the growth and metastasis of human neuroblastoma cells 35. Few studies have looked at the effects of soluble factors released by tumor cells in combination. The importance of investigating the diverse array of factors released by tumor cells (secretomes) is highlighted by the reality that antiangiogenic therapies targeting the inhibition of isolated molecules (e.g. VEGF) have often failed in mouse models of pancreatic neuroendocrine carcinoma and glioblastoma 36. In this regard, our study is the first detailed look at how TCM secretome affects tumor-related formation and leakage of blood vessels, along with imaging of tumor growth and spread in a CAM model of sarcoma.

First, our Multiplex analysis of the TCM secretome shows significant increases in angiogenesis-related cytokines 12 compared to the control medium. In the secretome of both U2OS and WEHI164 cell lines, three factors—FGF2, PDGF-AA, and VEGF—were notably enriched. In contrast, IL-8 and GM-CSF did not achieve statistical significance (Fig. 10A). Only U2OS secretome showed significantly higher levels of Epidermal Growth Factor (EGF), Transforming Growth Factor-alpha (TGF-α), Platelet-derived growth factor isoforms PDGF-AA, -BB (PDGF-AB/BB), Fractalkine, Macrophage-Derived Chemokine (MDC/CCL22), and growth-related oncogene α (GROα: CXCL1) than the control medium. These data posed the existence of both common and cell-type specific factors in TCM secretome. They play a critical role in the progression of metastasis in many cancers 37, including both prostate 38 and breast cancer^13^. Our research discovers that VEGF concentration in the secretome of the high-metastatic WEH164 cell line are 125 times higher than that of the low-metastatic U2OS cell line. This contrast implies that VEGF may greatly influence the metastatic behavior of WEH164 cells, suggesting its potential as a biomarker or therapeutic target in cancer research. Understanding VEGF’s role is vital for enhancing metastatic cancer treatments 39, prompting us to investigate its impacts on TCM-mediated OS tumor vascularization and metastasis in the CAM model.

As anticipated, Avastin^®^-mediated VEGF neutralization in TCM significantly abolish both VEGF expression and vascular leakage in tumor nodule tissues after U2OS cell implantation in CAM (Fig. 10B). Transient restoring of VEGF expression in these cells partly, but significantly, recovers both VEGF levels and blood-vessel leakage, confirming its effects in this in vivo model. Furthermore, Avastin’s VEGF neutralization in TCM significantly reduces neovascularization in the CAM tissue surrounding tumor nodules after U2OS cell implantation (Fig. 11A). Neutralizing VEGF significantly reduces tumor nodule volumes (Fig. 11B) and effectively eliminates metastases, as shown by whole embryo imaging (Fig. 11C). Again, restoring VEGF expression in these cells significantly improves CAM neovascularization, crucial for new blood vessel formation. This is linked to recovery of tumor nodule volumes and metastasis, highlighting the important role of VEGF in this model. In this context, several studies on osteosarcoma cohorts reveal the vital role of neo-vascularization markers in patient samples. In OS patients, amplification of VEGF pathway genes, particularly VEGF-A, has been reported and verified at the protein level 40. Elevated levels of VEGF are strongly correlated with the progression of tumor stages and the occurrence of metastasis 41,42. In metastatic patients, a marked increase in vascular density is a defining trait of primary OS tumors, unlike in non-metastatic patients 43. Hence, our findings emphasize the importance of VEGF in tumor-associated neovascularization, tumor growth and metastasis in CAM model of OS, confirming it could be a good target for the OS treatments.

## Limitations of current study

The CAM assay, despite challenges, has been successfully adapted for various tumors, as recently reviewed 44. However, the adaptation for sarcoma hasn’t been achieved yet, restricting the application of this promising in vivo model in research.

Our current study design could not exclude the fact that the TCM impact may be related to the synergistic effect between the repair mechanism after CAM abrasion and the active factors in TCM. In the CAM model, abrasion facilitated more rapid contact between tumor cells and the stromal layer or vascular network, and, triggers a series of natural repair responses in the CAM membrane, including angiogenesis and extracellular matrix (ECM) remodeling 45,46. Hence, more studies should be conducted to shed light on the effects of the repair system after CAM abrasion and the influencing factors in TCM.

Extracellular vesicles (EVs) are recognized as vital communicators among cells 47, especially in promoting angiogenesis in the relationship between OS and the bone microenvironment. It has been widely recognized that tumor-derived EV cargo significantly stimulates angiogenesis in various tumors 48. Adding tumor-conditioned medium, which contains exosomes (or EVs) and cytokines from sarcoma cells, increased tumor cell growth and promoted angiogenesis and ECM remodeling in vitro 49,50. Few recent studies found that OS-EVs play a pro-angiogenic role by carrying angiocrines and angiogenesis-related miRNA 51,52. While we concentrated on VEGFA in the current study, our TCM isolation method does not rule out the participation of other soluble pro-angiogenic factors and their carriers in the CAM model effects observed. Indeed, the transient VEGF overexpression in tumor cells did not completely reverse the loss of blood vessel formation, invasion, growth, and spread of sarcoma cells caused by VEGF-neutralizing monoclonal antibodies like bevacizumab (Avastin) (Figs. 10 and 11).

Behind vasculogenesis, vasculogenic mimicry (VM) might be crucial in developing a tumor’s vascular network 53. VM refers to a specific form of cancer cell’s plasticity that presents numerous implications. Insights into how aggressive cancer cells create vascular-mimic networks might offer new therapeutic strategies for tumor treatment. The main signaling molecules involved in VM are VEGF-VEGFR2 and PI3K-Akt-mTOR. These molecules regulate signals that boost blood vessel formation, permeability, tube formation, and endothelial marker expression 54. Osteosarcomas are profoundly vascularized tumors of the bone, developing in a hypoxic and acidic microenvironment. Despite this, the process of neovascularization in OS is still not clearly comprehended. In tumors, it is widely believed that the formation of new blood vessels from existing ones is the most important aspect of angiogenesis, but the role of endothelial progenitor cells (EPCs), which can develop into mature endothelial cells, must be noted in tumor vascular growth, even if there is less agreement on these cells’ involvement 55. An in vitro rat model study recently revealed that adding EPCs to encapsulated OS cells increased tumor blood vessel growth. In that model, the secretion of angiogenic factors (VEGF, TGF-β1, MCP-1, Activin A, and OPN) by OS rat tumor cells enhanced EPC migration and angiogenic properties^56^. While we have not yet examined the involvement of EPCs, our findings show that VEGF and TGF are enriched in the U2OS secretome (Fig. 10A). However, our research leaves open the possibility that TCM’s soluble factors might facilitate blood vessel development by activating chick EPCs or improving the vasculogenic mimicry of tumor stem-like cells in live subjects, warranting the need for additional experimental study.

## Materials and methods

### Cell culture

WEHI-164, mouse fibrosarcoma, and U2OS, human osteosarcoma, cell lines were obtained from Shared Research Facility “Vertebrate cell culture collection” (Institute of Cytology, RAS, Saint-Petersburg, Russia). Cell lines WEHI-164-Kat2S-T2A-Nluc, WEHI-164-Kat2S-T2A-Nluc-GFP and U2OS-Kat2S-T2A-Nluc, U2OS-Kat2S-T2A-Nluc-GFP were established in our laboratory. Cells were grown as described in Supplementary material (Methods, Sect.  1).

## Harvesting of TCM

Cells were cultured in T25 flasks until reaching 70–80% confluence. After that, the cell monolayers were thoroughly washed six times with HEPES buffer, prepared at 8.8 g/l and pH 7.4. Next, cultures were incubated in 10 ml of serum-free medium without phenol red for 48 h. The collected supernatant from cell monolayer was filtered through a 0.22 μm membrane filter to obtain cell-free tumor cell-conditioned medium (TCM). The TCM was subsequently concentrated using a 5 kDa molecular weight cutoff centrifugal filter unit (Jet Biofil, FTT405500,China) at 4 °C. Control medium, produced similarly, was concentrated and sterilized without contacting cells. (Detailed protocols can be found in the Supplementary Method, Sect.  2).

### Preparation of dual-reporter cell lines

#### Plasmids

We created the PLKO-Katushka2S-T2A-Nluc plasmid to set up a dual-reporter system that co-expresses the Katushka2S far-red fluorescent protein and NanoLuc luciferase (Nluc). Detailed cloning procedures and primer sequences are provided in Supplementary (Methods, Sect.  3 Supplementary Fig. 1 and Table 1, respectively).

For VEGFA overexpression, we used the plasmid FC-3398 hVEGFA-HA-OE (6,605 bp), which encodes human VEGFA under the control of a CMV promoter (Fubio Biotechnology Co., Ltd., Suzhou, China).The plasmid map is presented in Supplementary Fig. 2.

### Lentivirus production and transduction

Virus was packaged and harvested in HEK293T (ATCC) cells using pLP1 (encoding HIV-1 Gag and Pol proteins), pVSVG (encoding the VSV G envelope protein), pLP2 (encoding HIV-1 Rev proteins), and Katushka 2 S-T2A-Nluc (or PLKO-3G) vectors as previously described 57. Two milliliters of the lentiviral supernatant were added to tumor cells, and the transduced cells were sorted out using a cell sorter (BIO-RAD S3e).

### Optimization of CAM model of sarcoma

The CAM’s highly vascularized structure makes it an excellent platform for studying both experimental and spontaneous metastasis (Fig. 6), allowing detailed investigations into the complex tumor metastatic process^44^,^58^.

### TCM pre-treatment in the spontaneous metastasis CAM model

Fertilized, specific pathogen free (SPF) eggs for the experiment were purchased from a local ecological hatchery (Trade house Ptichnoe, Ltd., https://ptichnoe-td.ru), and were incubated 37 °C with 70% relative humidity, and proceeded as described previously 59. The entire procedure of TCM-pre-treated CAM (TCM-CAM) assay is described in Supplementary (Methods, Sect.  4) and is depicted on Fig. 4.

**The experimental metastasis CAM model.**

For tumor cell injection experiments, the protocol was adapted from the experimental procedure described by Trenis D Palmer et al.^60^ and performed on EMD12. For both cell lines, the number of injected cells was the same as in Sect.  4. A 100 µl cell suspension was injected into the allantoic vein of each embryo using a 30-gauge syringe. Eggs were collected on EMD16.

### Tumor nodule tissue processing

Embryos were frozen on EMD16. Subsequently, the opening was enlarged, and the tumor was carefully resected along the O-ring. The O-rings and excess CAM tissue were removed in PBS. Tumor tissues were immersed in 4% paraformaldehyde and fixed overnight at 4 °C. Chicken embryos were also collected in 100-mm plates for metastasis imaging. The tumor diameter and height were measured as previously described^61^. Macroscopic images of the tumors were acquired using a Leica M60 microscope.

### Fluorescent and luminescent bioimaging

Bioimaging was performed using LumoTrace^®^ Fluo (Abisense LLC, Russia) imaging system. Katushka 2 S signals were acquired with 590 nm excitation wavelength (LED) and a 655 nm longpass emission filter. GFP signals were imaged with 470 nm excitation wavelength (LED) and a 550 nm longpass emission filter. For both Katushka2S and GFP imaging, the LED illumination angle was set to 80 degrees, and the exposure time was 5000 ms.

The substrate furimazine (5 µg in 100 µl)^62^ was injected into tissues or organs under dark-field conditions using a 30G needle to detect Luciferase signals. Imaging parameters included an exposure time of 10–20 s and image acquisition with 8 × 8 binning. Experimental data were processed and analyzed using Icy software (https://icy.bioimageanalysis.org/).

### Immuno-fluorescent analysis

Tissue processing and sectioning for immunofluorescent staining was performed as we previously described^63^ (Supplementary Method, Sect.  5). Staining with primary and secondary antibodies (see Supplementary Table 2) were done as we published previously^64^. Fluorescence imaging of sections was accomplished with the EVOS™ M5000 Imaging System.

### Angiogenesis assay

As described in (Supplementary Method, Sect.  4), following windowing and pre-treaing with either the TCM or SFM (using serum-free antibiotic-containing medium), the vascular density of CAMs was recorded by using a portable microscope (TOMLOV, DM601, USA) placed over the O-rings (noted as hour 0). All CAM groups were photographed to record neo-angiogenesis at 72 h after CAM treatment. Tumors within the O-ring were excised at EMD12 by adding 2 ml of fixative (methanol: acetone 1:1) on CAM for 15 min and photographed using a Leica M60 microscope 65. Quantification of vessel density was performed using the Vessel Analysis plugin(https://imagej.net/plugins/vessel-analysis) implemented in ImageJ software (National Institute of Health, USA).

### Multiplex Assay

A comprehensive analysis of 41 distinct cytokine and chemokine biomarkers derived from both the Control medium and TCM obtained from each individual cell line was accomplished using the Bead-Based Multiplex Assay (MILLIPLEX MAP Human Cytokine/Chemokine Magnetic Bead Panel - Premixed 41 Plex, cat. # HCYTMAG-60 K-PX41, Millipore, Merck Russia) and Luminex technology with the QuattroPlex biomarker analysis system (QuattroPlexLab_comp, DIA-M, Russia). (Supplementary Methods, Sect.  6)

### Vascular leakage assay

To assess tumor-associated vascular permeability, a 70 kDa FITC–dextran solution (Sigma-Aldrich) was intravenously injected into the CAM vasculature on day 8 after tumor cell implantation (EMD16). Following incubation at 37 °C for 30 min, embryos were frozen and processed and evaluated according to previously published protocol^66^. Image analysis was performed using ImageJ software to quantify the total area of FITC fluorescence within the tumor region as an indicator of vascular permeability. All imaging parameters were kept consistent across experimental groups. For detailed procedures, please refer to the Supplementary Methods, Sect.  8.

### Neutralization of VEGF in TCM

To neutralize VEGFA in tumor-conditioned media (TCM), a clinically approved monoclonal antibody, bevacizumab (Avastin^®^, 25 mg/ml, 100 mg/4 ml, concentrate for i.v. injection, Roche-Moscow, Russia), was added to both the experimental and rescue group TCMs at a final concentration of 20 ng/ml ^67,68^, following filtration as described in Sect.  8.

### Statistical analysis

All data are presented as mean values ± standard error of the mean (SEM). Statistical significance was assessed using GraphPad Prism software (version 10.0). For comparisons between two groups, the nonparametric Mann–Whitney test was applied, and Pearson correlation analysis was used where appropriate. For comparisons among multiple groups, one-way ANOVA or two-way ANOVA was performed as indicated. Statistical significance is denoted as follows: ns, *P* > 0.05; *P < 0.05; **P < 0.01; ***P < 0.001.

## Supplementary Information

Below is the link to the electronic supplementary material.

Supplementary Material 1

## Data Availability

The datasets used and/or analysed during the current study available from the corresponding author on reasonable request.The bio-imaging data that support the findings of this study are available from Abisense LLC but restrictions apply to the availability of these data, which were used under license for the current study, and so are not publicly available. Data are however available from the authors upon reasonable request and with permission of Abisense LLC.

## References

[1] Bleloch, J. S. et al. Managing sarcoma: where have we come from and where are we going? *Therapeutic Adv. Med. Oncol.* **9**, 637–659. 10.1177/1758834017728927 (2017). 10.1177/1758834017728927 PMC5613860 28974986

[2] Damron, T. A., Ward, W. G. & Stewart, A. Osteosarcoma, chondrosarcoma, and Ewing’s sarcoma: national cancer data base report. *Clin. Orthop. Relat. Research*. **459**, 40–47. 10.1097/BLO.0b013e318059b8c9 (2007). 10.1097/BLO.0b013e318059b8c9 17414166

[3] Beird, H. C. et al. Osteosarcoma. *Nat. reviews Disease primers*. **8**, 77. 10.1038/s41572-022-00409-y (2022). 36481668 10.1038/s41572-022-00409-y

[4] Ferreira, C. R., da Fonseca, L. G., Piotto, G. H. M., Geyer, F. C. & de Alcântara, P. S. M. Fibrosarcoma: a challenging diagnosis. *Autopsy Case Rep.* **3**, 21. 10.4322/acr.2013.024 (2013). 10.4322/acr.2013.024 PMC6671889 31528615

[5] Vanni, S. et al. Myxofibrosarcoma landscape: Diagnostic pitfalls, clinical management and future perspectives. *Therapeutic Adv. Med. Oncol.* **14**, 17588359221093973. 10.1177/17588359221093973 (2022). 10.1177/17588359221093973 PMC9244941 35782752

[6] Kunz, P., Schenker, A., Sähr, H., Lehner, B. & Fellenberg, J. Optimization of the chicken chorioallantoic membrane assay as reliable in vivo model for the analysis of osteosarcoma. *PLoS One*. **14**, e0215312. 10.1371/journal.pone.0215312 (2019). 30986223 10.1371/journal.pone.0215312 PMC6464229

[7] Ribatti, D. The chick embryo chorioallantoic membrane as an experimental model to study in vivo angiogenesis in glioblastoma multiforme. *Brain Res. Bull.* **182**, 26–29. 10.1016/j.brainresbull.2022.02.005 (2022). 35143927 10.1016/j.brainresbull.2022.02.005

[8] Rovithi, M. et al. Development of bioluminescent chick chorioallantoic membrane (CAM) models for primary pancreatic cancer cells: a platform for drug testing. *Sci. Rep.* **7**, 44686. 10.1038/srep44686 (2017). 28304379 10.1038/srep44686 PMC5356332

[9] Xiao, L. et al. RORα inhibits adipocyte-conditioned medium-induced colorectal cancer cell proliferation and migration and chick embryo chorioallantoic membrane angiopoiesis. *Am. J. Physiology-Cell Physiol.* **308**, C385–C396. 10.1152/ajpcell.00091.2014 (2015). 10.1152/ajpcell.00091.2014 25500738

[10] Laborda-Illanes, A. et al. Development of in vitro and in vivo tools to evaluate the antiangiogenic potential of melatonin to neutralize the angiogenic effects of VEGF and breast cancer cells: CAM assay and 3D endothelial cell spheroids. *Biomed. Pharmacother.* **157**, 114041. 10.1016/j.biopha.2022.114041 (2023). 36423543 10.1016/j.biopha.2022.114041

[11] Rabas, N., Ferreira, R. M., Di Blasio, S. & Malanchi, I. Cancer-induced systemic pre-conditioning of distant organs: building a niche for metastatic cells. *Nat. Rev. Cancer*. **24**, 829–849. 10.1038/s41568-024-00752-0 (2024). 39390247 10.1038/s41568-024-00752-0

[12] Ritchie, S., Reed, D. A., Pereira, B. A. & Timpson, P. The cancer cell secretome drives cooperative manipulation of the tumour microenvironment to accelerate tumourigenesis. *Faculty reviews* 10, 4, (2021). 10.1186/s40001-024-01711-z 10.12703/r/10-4 PMC7894270 33659922

[13] Zahari, S., Syafruddin, S. E. & Mohtar, M. A. Impact of the Cancer Cell Secretome in driving breast Cancer progression. *Cancers* **15**, 2653. 10.3390/cancers15092653 (2023). 37174117 10.3390/cancers15092653 PMC10177134

[14] Nakano, K. Challenges of systemic therapy investigations for bone sarcomas. *Int. J. Mol. Sci.* **23**, 3540. 10.3390/ijms23073540 (2022). 35408900 10.3390/ijms23073540 PMC8998654

[15] Blay, J. Y. et al. Synovial sarcoma: characteristics, challenges, and evolving therapeutic strategies. *ESMO open.* **8**, 101618. 10.1016/j.esmoop.2023.101618 (2023). 37625194 10.1016/j.esmoop.2023.101618 PMC10470271

[16] Hingorani, P. et al. Current state of pediatric sarcoma biology and opportunities for future discovery: A report from the sarcoma translational research workshop. *Cancer Genet.* **209**, 182–194. 10.1016/j.cancergen.2016.03.004 (2016). 27132463 10.1016/j.cancergen.2016.03.004 PMC5497490

[17] Jeong, S. et al. Sarcoma Immunotherapy: Confronting Present Hurdles and Unveiling Upcoming Opportunities. *Mol. Cells*. **46**, 579–588. 10.14348/molcells.2023.0079 (2023). 37853684 10.14348/molcells.2023.0079 PMC10590708

[18] Blocker, S. J. et al. MR histology reveals tissue features beneath heterogeneous MRI signal in genetically engineered mouse models of sarcoma. *Front. Oncol.* **14**, 1287479. 10.3389/fonc.2024.1287479 (2024). 38884083 10.3389/fonc.2024.1287479 PMC11176416

[19] Patel, R. et al. Neoadjuvant radiation therapy and surgery improves metastasis-free survival over surgery alone in a primary mouse model of soft tissue sarcoma. *Mol. Cancer Ther.* **22**, 112–122. 10.1158/1535-7163.MCT-21-0991 (2023). 36162051 10.1158/1535-7163.MCT-21-0991 PMC9812921

[20] Sorimachi, Y. et al. Mesenchymal loss of p53 alters stem cell capacity and models human soft tissue sarcoma traits. *Stem Cell. Rep.* **18**, 1211–1226. 10.1016/j.stemcr.2023.03.009 (2023). 10.1016/j.stemcr.2023.03.009 PMC10202654 37059101

[21] Fischer, D. et al. The CAM model—Q&A with experts. *Cancers* **15**, 191. 10.3390/cancers15010191 (2022). 36612187 10.3390/cancers15010191 PMC9818221

[22] Merckx, G. et al. Chorioallantoic membrane assay as model for angiogenesis in tissue engineering: Focus on stem cells. *Tissue Eng. Part. B: Reviews*. **26**, 519–539. 10.1089/ten.teb.2020.0048 (2020). 10.1089/ten.TEB.2020.0048 32220219

[23] Mapanao, A. K. et al. Tumor grafted–chick chorioallantoic membrane as an alternative model for biological cancer research and conventional/nanomaterial-based theranostics evaluation. *Expert Opin. Drug Metab. Toxicol.* **17**, 947–968. 10.1080/17425255.2021.1879047 (2021). 33565346 10.1080/17425255.2021.1879047

[24] Schneider-Stock, R. & Ribatti, D. The CAM assay as an alternative in vivo model for drug testing. *Organotypic Models Drug Dev.* 303–323. 10.1007/164_2020_375 (2021). 10.1007/164_2020_375 32776283

[25] Pawlikowska, P. et al. Exploitation of the chick embryo chorioallantoic membrane (CAM) as a platform for anti-metastatic drug testing. *Sci. Rep.* **10**, 16876. 10.1038/s41598-020-73632-w (2020). 33037240 10.1038/s41598-020-73632-w PMC7547099

[26] Sys, G. et al. Tumor grafts derived from sarcoma patients retain tumor morphology, viability, and invasion potential and indicate disease outcomes in the chick chorioallantoic membrane model. *Cancer Lett.* **326**, 69–78. 10.1016/j.canlet.2012.07.023 (2012). 22841668 10.1016/j.canlet.2012.07.023

[27] Sys, G. M. et al. The in ovo CAM-assay as a xenograft model for sarcoma. *J. visualized experiments: JoVE*. 50522. 10.3791/50522 (2013). 10.3791/50522 PMC3845689 23892612

[28] Nogawa, M. et al. Monitoring luciferase-labeled cancer cell growth and metastasis in different in vivo models. *Cancer Lett.* **217**, 243–253. 10.1016/j.canlet.2004.07.010 (2005). 15617843 10.1016/j.canlet.2004.07.010

[29] Dvorak, H., Nagy, J., Feng, D., Brown, L. & Dvorak, A. Vascular permeability factor/vascular endothelial growth factor and the significance of microvascular hyperpermeability in angiogenesis. *Vascular growth factors angiogenesis*. 97–132. 10.1007/978-3-642-59953-8_6 (1999). 10.1007/978-3-642-59953-8_6 9893348

[30] Lamalice, L., Le Boeuf, F. & Huot, J. Endothelial cell migration during angiogenesis. *Circul. Res.* **100**, 782–794. 10.1161/01.RES.0000259593.07661.1e (2007). 10.1161/01.RES.0000259593.07661.1e 17395884

[31] Matsuda, Y., Hagio, M. & Ishiwata, T. Nestin: a novel angiogenesis marker and possible target for tumor angiogenesis. *World J. gastroenterology: WJG*. **19**, 42. 10.3748/wjg.v19.i1.42 (2013). 10.3748/wjg.v19.i1.42 PMC3545228 23326161

[32] Liu, Z. L., Chen, H. H., Zheng, L. L., Sun, L. P. & Shi, L. Angiogenic signaling pathways and anti-angiogenic therapy for cancer. *Signal. Transduct. Target. therapy*. **8**, 198. 10.1038/s41392-023-01460-1 (2023). 10.1038/s41392-023-01460-1 PMC10175505 37169756

[33] Tufan, A. C. & Satiroglu-Tufan, N. L. The chick embryo chorioallantoic membrane as a model system for the study of tumor angiogenesis, invasion and development of anti-angiogenic agents. *Curr. Cancer Drug Targets*. **5**, 249–266. 10.2174/1568009054064624 (2005). 15975046 10.2174/1568009054064624

[34] Ribatti, D. Chick embryo chorioallantoic membrane as a useful tool to study angiogenesis. *Int. Rev. cell. Mol. biology*. **270**, 181–224. 10.1016/S1937-6448(08)01405-6 (2008). 10.1016/S1937-6448(08)01405-6 19081537

[35] Brignole, C. et al. Effect of bortezomib on human neuroblastoma cell growth, apoptosis, and angiogenesis. *J. Natl Cancer Inst.* **98**, 1142–1157. 10.1093/jnci/djj309 (2006). 16912267 10.1093/jnci/djj309

[36] Pàez-Ribes, M. et al. Antiangiogenic therapy elicits malignant progression of tumors to increased local invasion and distant metastasis. *Cancer cell.* **15**, 220–231. 10.1016/j.ccr.2009.01.027 (2009). 19249680 10.1016/j.ccr.2009.01.027 PMC2874829

[37] Qu, R., Zhao, Y. & Zhang, Y. The mechanism of cytokine regulation of cancer occurrence and development in the tumor microenvironment and its application in cancer treatment: a narrative review. *Translational Cancer Res.* **13**, 5649. 10.21037/tcr-24-679 (2024). 10.21037/tcr-24-679 PMC11543031 39525000

[38] Adekoya, T. O. & Richardson, R. M. Cytokines and chemokines as mediators of prostate cancer metastasis. *Int. J. Mol. Sci.* **21**, 4449. 10.3390/ijms21124449 (2020). 32585812 10.3390/ijms21124449 PMC7352203

[39] Guelfi, S., Hodivala-Dilke, K. & Bergers, G. Targeting the tumour vasculature: from vessel destruction to promotion. *Nat. Rev. Cancer*. **24**, 655–675. 10.1038/s41568-024-00736-0 (2024). 39210063 10.1038/s41568-024-00736-0

[40] Yang, J. et al. Genetic amplification of the vascular endothelial growth factor (VEGF) pathway genes, including VEGFA, in human osteosarcoma. *Cancer* **117**, 4925–4938. 10.1002/cncr.26116 (2011). 21495021 10.1002/cncr.26116 PMC3465081

[41] Lammli, J. et al. Expression of Vascular Endothelial Growth Factor correlates with the advance of clinical osteosarcoma. *Int. Orthop.* **36**, 2307–2313. 10.1007/s00264-012-1629-z (2012). 22855059 10.1007/s00264-012-1629-z PMC3479297

[42] Kaya, M. et al. Vascular endothelial growth factor expression in untreated osteosarcoma is predictive of pulmonary metastasis and poor prognosis. *Clin. Cancer Res.* **6**, 572–577 (2000). 10690541

[43] Dumars, C. et al. Dysregulation of macrophage polarization is associated with the metastatic process in osteosarcoma. *Oncotarget* **7**, 78343. 10.18632/oncotarget.13055 (2016). 27823976 10.18632/oncotarget.13055 PMC5346643

[44] Wang, Y., Xue, W., Pustovalova, M., Kuzmin, D. V. & Leonov, S. Chick Embryo Chorioallantoic Membrane (CAM) Model for Cancer Studies and Drug Evaluation. *Front. Bioscience-Landmark*. **30**, 37456. 10.31083/FBL37456 (2025). 10.31083/FBL37456 40464506

[45] Ribatti, D. *The Chick Embryo Chorioallantoic Membrane in the Study of Angiogenesis and Metastasis: The CAM assay in the study of angiogenesis and metastasis* (Springer Science & Business Media, 2010).

[46] Deryugina, E. I. & Quigley, J. P. Chick embryo chorioallantoic membrane models to quantify angiogenesis induced by inflammatory and tumor cells or purified effector molecules. *Methods Enzymol.* **444**, 21–41. 10.1016/S0076-6879(08)02802-4 (2008). 19007659 10.1016/S0076-6879(08)02802-4 PMC2699944

[47] Mathieu, M., Martin-Jaular, L., Lavieu, G. & Théry, C. Specificities of secretion and uptake of exosomes and other extracellular vesicles for cell-to-cell communication. *Nat. Cell Biol.* **21**, 9–17. 10.1038/s41556-018-0250-9 (2019). 30602770 10.1038/s41556-018-0250-9

[48] Aslan, C. et al. Tumor-derived exosomes: Implication in angiogenesis and antiangiogenesis cancer therapy. *J. Cell. Physiol.* **234**, 16885–16903. 10.1002/jcp.28374 (2019). 30793767 10.1002/jcp.28374

[49] Nairon, K. G., DePalma, T. J., Zent, J. M., Leight, J. L. & Skardal, A. Tumor cell-conditioned media drives collagen remodeling via fibroblast and pericyte activation in an in vitro premetastatic niche model. *IScience* 25, (2022). 10.1016/j.isci.2022.104645 PMC9257340 35811850

[50] Oliinyk, D., Eigenberger, A., Felthaus, O., Haerteis, S. & Prantl, L. Chorioallantoic membrane assay at the cross-roads of adipose-tissue-derived stem cell research. *Cells* **12**, 592. 10.3390/cells12040592 (2023). 36831259 10.3390/cells12040592 PMC9953848

[51] Perut, F., Roncuzzi, L., Zini, N., Massa, A. & Baldini, N. Extracellular nanovesicles secreted by human osteosarcoma cells promote angiogenesis. *Cancers* **11**, 779. 10.3390/cancers11060779 (2019). 31195680 10.3390/cancers11060779 PMC6627280

[52] Raimondi, L. et al. Osteosarcoma cell-derived exosomes affect tumor microenvironment by specific packaging of microRNAs. *Carcinogenesis* **41**, 666–677. 10.1093/carcin/bgz130 (2020). 31294446 10.1093/carcin/bgz130

[53] Carmeliet, P. & Jain, R. K. Molecular mechanisms and clinical applications of angiogenesis. *Nature* **473**, 298–307. 10.1038/nature10144 (2011). 21593862 10.1038/nature10144 PMC4049445

[54] Murai, T. & Matsuda, S. Targeting the PI3K-Akt-mTOR signaling pathway involved in vasculogenic mimicry promoted by cancer stem cells. *Am. J. Cancer Res.* **13**, 5039 (2023). 38058805 PMC10695779

[55] Marçola, M. & Rodrigues, C. E. Endothelial progenitor cells in tumor angiogenesis: another brick in the wall. *Stem cells international* 832649, (2015). 10.1155/2015/832649 (2015). 10.1155/2015/832649 PMC4427119 26000021

[56] An, R. et al. Proangiogenic effects of tumor cells on endothelial progenitor cells vary with tumor type in an in vitro and in vivo rat model. *FASEB J.* **32**, 5587–5601. 10.1096/fj.201800135RR (2018). 29746168 10.1096/fj.201800135RR

[57] Tiscornia, G., Singer, O. & Verma, I. M. Production and purification of lentiviral vectors. *Nat. Protoc.* **1**, 241–245. 10.1038/nprot.2006.37 (2006). 17406239 10.1038/nprot.2006.37

[58] Palmer, T. D., Lewis, J. & Zijlstra, A. Quantitative analysis of cancer metastasis using an avian embryo model. *J. visualized experiments: JoVE*. 2815. 10.3791/2815 (2011). 10.3791/2815 PMC3197130 21673636

[59] Nowak-Sliwinska, P., Segura, T. & Iruela-Arispe, M. L. The chicken chorioallantoic membrane model in biology, medicine and bioengineering. *Angiogenesis* **17**, 779–804. 10.1007/s10456-014-9440-7 (2014). 25138280 10.1007/s10456-014-9440-7 PMC4583126

[60] Palmer, T. D., Lewis, J. & Zijlstra, A. Quantitative analysis of cancer metastasis using an avian embryo model. *JoVE (Journal Visualized Experiments)*. e2815. 10.3791/2815 (2011). 10.3791/2815 PMC3197130 21673636

[61] Richtig, E., Langmann, G., Müllner, K., Richtig, G. & Smolle, J. Calculated tumour volume as a prognostic parameter for survival in choroidal melanomas. *Eye* **18**, 619–623. 10.1038/sj.eye.6700720 (2004). 15184927 10.1038/sj.eye.6700720

[62] Stacer, A. C. et al. NanoLuc reporter for dual luciferase imaging in living animals. *Molecular imaging* **12**, 7290. 00062, (2013). 10.2310/7290.2013.00062 (2013). PMC4144862 24371848

[63] Sakr, N. et al. Characterizing and quenching autofluorescence in fixed mouse adrenal cortex tissue. *Int. J. Mol. Sci.* **24**, 3432. 10.3390/ijms24043432 (2023). 36834842 10.3390/ijms24043432 PMC9968082

[64] Pustovalova, M. et al. CD44 + and CD133 + non-small cell lung cancer cells exhibit DNA damage response pathways and dormant polyploid giant cancer cell enrichment relating to their p53 status. *Int. J. Mol. Sci.* **23**, 4922. 10.3390/ijms23094922 (2022). 35563313 10.3390/ijms23094922 PMC9101266

[65] Guo, X. et al. RUNX1 promotes angiogenesis in colorectal cancer by regulating the crosstalk between tumor cells and tumor associated macrophages. *Biomark. Res.* **12**, 29. 10.1186/s40364-024-00573-1 (2024). 38419056 10.1186/s40364-024-00573-1 PMC10903076

[66] Qin, W. et al. Bacteria-elicited specific thrombosis utilizing acid‐induced cytolysin A expression to enable potent tumor therapy. *Adv. Sci.* **9**, 2105086. 10.1002/advs.202105086 (2022). 10.1002/advs.202105086 PMC9130894 35411710

[67] Comşa, Ş. et al. Bevacizumab modulation of the interaction between the MCF-7 cell line and the chick embryo chorioallantoic membrane. *vivo* **31**, 199–203. 10.21873/invivo.11045 (2017). 10.21873/invivo.11045 PMC5411745 28358700

[68] Shinde, D. *Chicken embryo chorioallantoic membrane assay for pre-clinical evaluation of efficacy and safety of anti-angiogenic and hypoxic cell-starving tumor interventions* (University of Zurich, 2014).

