# Supplementary material for: A CAM bioimaging model reveals the connection between VEGFA vascular remodeling and enhanced sarcoma progression via tumor secretome
Source: Sci Rep. 2026 Mar 7;16:12525. doi: 10.1038/s41598-026-42154-2 (PMC13087146; doi:10.1038/s41598-026-42154-2)
Supplement: Supplementary file 1 — Supplementary Material 1 [file 41598_2026_42154_MOESM1_ESM.docx]

**Supplementary Information**

# Methods

**1.Cell culture**

WEHI-164, mouse fibrosarcoma, and U2OS, human osteosarcoma, cell lines were obtained from Shared Research Facility “Vertebrate cell culture collection” (Institute of Cytology, RAS, Saint-Petersburg, Russia). Cell lines WEHI-164-Kat2S-T2A-Nluc, WEHI-164-Kat2S-T2A-Nluc-GFP and U2OS-Kat2S-T2A-Nluc, U2OS-Kat2S-T2A-Nluc-GFP were established in our laboratory. Cells were grown as described in[1, 2]. All the cell lines were cultured in DMEM (NPP PanEko LLC, Moscow, Russia) supplemented with 10% fetal bovine serum and 1% penicillin-streptomycin (5000 U/mL) , 1% glutamine. Both of the cells were maintained in the humidified incubator under the condition 37$℃$ temperatures, 5% CO_2_. The medium was refreshed two times per week. All cell lines were validated by STR profiling and tested negative for mycoplasma (MycoReport Mycoplasma Detection Kit, Cat. # MR001, Evrogene, Russia).

**2.Harvesting of TCM**

For the preparation of TCM, cells were cultured in T25 flasks until reaching 70-80% confluence. After that, the cell monolayers were thoroughly washed six times with HEPES buffer, prepared at 8.8 g/l and pH 7.4. This careful washing step was essential for completely removing any leftover serum components that might affect later tests. Proper handling of the buffer solution and repeated washing emphasized the need for a clean environment for cell cultures. After this process, the monolayers were deemed ready for the next phase of experimentation. Next, cultures were incubated in 10 ml of serum-free medium without phenol red for 48 h. The collected supernatant from cell monolayer was filtered through a 0.22 μm membrane filter to obtain cell-free tumor cell-conditioned medium (TCM). The TCM was subsequently concentrated using a 5 kDa molecular weight cutoff centrifugal filter unit (Jet Biofil, FTT105500, China) at 4°C. This was done by centrifuging at 4,000 × g until the concentrate volume was reduced to 1/10 to 1/50 of the original volume. Control medium, produced similarly, was concentrated and sterilized without contacting cells.

**3.Plasmids**

pKatushka2S-C (Cat.# FP761), a mammalian expression vector encoding far-red fluorescent protein Katushka2S, was obtained from Evrogen (Russia). The vector allows generation of fusions to the Katushka2S C-terminus and expression of Katushka2S fusions or Katushka2S alone in eukaryotic (mammalian) cells. The pCDH-EF1-N-cG-Neo plasmid was from Addgene (Plasmid #124978). The construct pKatushka 2S-T2A-Nluc was generated in the following way. The PCR fragment Nluc, which contains the Nluc gene open reading frame (ORF), was amplified from the pCDH-EF1-N-cG-Neo plasmid with primers Nluc-Dir and EcoRI-Nluc-Rev (Supplementary Fig. 1A). The PCR fragment T2A-Nluc was created through an assembly PCR of the PCR fragment Nluc combined with oligonucleotides T2A-Oligo-1 and T2A-Oligo-2 applying primers XhoI-T2A-Dir and EcoRI-Nluc-Rev (Supplementary Fig. 1B).

The resulting The PCR fragment T2A-Nluc was digested with restriction enzymes XhoI and EcoRI and cloned in XhoI and EcoRI restriction sites of the vector pKatushka2S creating ORF for a new reporter gene Katushka 2S-T2A-Nluc. The resulting construct was verified by the Sanger sequencing and designated pKatushka 2S-T2A-Nluc. The Katushka 2S-T2A-Nluc ORF was then amplified with primers Kat-T2A-Nluc-Dir and Kat-T2A-Nluc-Rev, digestated with restriction enzymes BamHI and EcoRV and cloned in the restriction sites BamHI and EcoRV of the lentiviral vector PLKO.3G vector (Addgene) instead of the EGFP ORF, generating lentiviral construct PLKO-Katushka 2S-T2A-Nluc. A list of used primers and oligonucleotides can be found in Supplementary Table 1.

A mammalian expression plasmid, FC-3398 hVEGFA-HA-OE (6,605 bp), encoding human VEGFA under the control of a CMV promoter, was obtained from Fubio Biotechnology Co., Ltd. (Suzhou, China). This vector also contains an SV40 origin, a Neomycin resistance gene for selection, and a bGH polyadenylation signal to ensure transcript stability. The plasmid map is presented in Supplementary Fig. 2.

**4.TCM pre-treatment in the spontaneous metastasis CAM model**

Fertilized, specific pathogen free (SPF) eggs for the experiment were purchased from a local ecological hatchery (Trade house Ptichnoe, Ltd., <https://ptichnoe-td.ru>), and were incubated 37 °C with 70% relative humidity, and proceeded as described previously [3]. Briefly, this day was designated as chicken embryo development day 0 (EMD 0). On EMD 3-4, a window can be opened once radial vessels appear on the CAM. It can be observed under an egg candler. To minimize contamination of chicken embryos, all procedures were conducted within a laminar flow hood under sterile conditions. Using an egg candler, the locations of embryonic contact points, air sacs, and major blood vessels were marked on the eggshell with a pencil. About 2-3 ml of egg white was then withdrawn using an 18G needle inserted at the lower portion of the blunt end of the egg. The needle puncture was subsequently sealed with a semi-permeable adhesive film (3M film). A circle with an approximate diameter of two centimeters was drawn at the region where the CAM had descended, and a window was then created using a mini chainsaw. Following this, the window was sealed with a semi-permeable adhesive film(3M film) and the eggs were placed horizontally back into the incubator. On chicken embryo development day 7 (EMD7), an autoclaved Teflon ring (PTFE O-ring, inner diameter 6 mm, outer diameter 9 mm) was first placed on the CAM surface to define the treatment area. Subsequently, minimal mechanical injury was introduced by gently tapping the CAM surface once or twice within the ring using a sterile disposable cotton swab, until a small pinpoint bleeding was observed. This procedure was performed without any dragging motion or repeated scratching to minimize tissue disruption. Following minimal scratching, 50 µl of tumor-conditioned medium (TCM) was applied onto the CAM surface within the enclosed area. The CAM surface was then gently pierced with a 30G needle, after which 100 µl of TCM was injected into the CAM mesoderm within the ringed area. Cell seeding was performed on EMD8, with cells seeded inside the Teflon rings. For the WEHI-164 and U2OS cell lines, the optimal seeding density was 1.5 x 10^6^ and 3 x 10^6^ cells in 20 µl conditioned medium, respectively. Following cell seeding, the window was resealed with semi-permeable adhesive film (3M film). On EMD16, the tumors were harvested and processed for bioimaging.

**5. The experimental metastasis CAM model.**

For tumor cell injection experiments, the protocol was adapted from the experimental procedure described by Trenis D Palmer et al.[4]. On EMD12, eggs were positioned horizontally on an egg rack and illuminated with an egg candler to determine the location of the air sac at the blunt end. The chorioallantoic vein was firstly identified and marked, and then a circle with 1 cm diameter was drawn on the eggshell surrounding the vein. A window was subsequently created in the marked area using a mini chainsaw, exposing the eggshell membrane, which was made transparent using mineral oil. Tumor cells were dissociated from the culture dish, washed with ten volumes of phosphate-buffered saline (PBS), centrifuged, and resuspended in 100 µl of serum-free medium. For both cell lines, the number of injected cells was the same as in section 2.3.1. A 100 µl cell suspension was injected into the allantoic vein of each embryo using a 30-gauge syringe. Eggs were collected on EMD16.

**6.Immuno-fluorescent analysis**

For tissue immunocytochemical staining, tissues were initially dehydrated in a 15% sucrose solution and incubated overnight at 4°C. The solution was then replaced with Surcrose 30%, and tissues were incubated at 4°C for an additional days. The tissues were subsequently embedded in Tissue Plus® OCT compound(Fisher Healthcare Tissue-Plus™ O.C.T. Compound) and snap-frozen in liquid nitrogen. Tumors were subsequently sectioned at 12 µm thickness using a cryotome (Cryotome FSE, Thermo Fisher Scientific). Before starting the experiment, tissue slides were thawed at room temperature for 20 minutes, followed by a 10-minute wash with PBS to remove Tissue Plus® OCT compound. Membrane permeabilization was performed using 0.5% Triton X-100, followed by 2-3 washes with PBS, and the slides were carefully dried from the edges with blotting paper. Approximately 200 µl of blocking solution (10% goat serum, 0.25% Triton X-100, 0.25% Tween-20, 0.1% sodium citrate, 0.3 M glycine, and 1% BSA) was added to cover the tissue, and slides were incubated for 2-3 hours. After drying the blocking solution, the primary antibodies (Ki-67 Rat Monoclonal Antibody (SolA15), eBioscience™, ThermoFisher scientific; Anti-VEGFA Rabbit Monoclonal Antibody (GB15165-100), ServiceBio Inc.) were diluted to 5 µg/mL in staining buffer (0.25% Triton X-100, 0.25% Tween-20, and 1% BSA), and 200 µl was added to each slide for the experimental group. For the control group, non-immune rat or rabbit IgG in 200 µl of staining buffer was added. All slides were incubated at 4°C overnight. On the following day, slides were washed for 10 minutes with washing buffer (0.1% Triton X-100 and 0.1% Tween-20). The secondary antibodies (Goat either Anti-Rat or Anti-Rabbit IgG H&L, conjugated with Alexa Fluor®647, for cell line with GFP protein reporting; Goat either Anti-Rat or Anti-Rabbit IgG H&L, conjugated with Alexa Fluor®488 for cell line without GFP protein reporter transfection) were diluted 1:400 in washing buffer, and 200 µl of the diluted solution was added to each slide, followed by incubation for 1.5 hours. Samples were then washed twice with fresh washing buffer for 10 minutes each, incubated with 0.5 µg/mL DAPI solution for 5-10 minutes at room temperature, and subsequently washed with PBS. Finally, mounting solution (VECTASHIELD® PLUS Antifade Mounting Medium, Vector Laboratories, Cat.# № H-1000-10, CHIMMED Ltd., Russia) was dropped on the sections, then sections were sealed with coverslips. Fluorescence imaging of sections was accomplished with the EVOS™ M5000 Imaging System.

**7.Multiplex Assay**

To thoroughly evaluate the secretome constituents, a comprehensive analysis was conducted focusing on 41 distinct cytokine and chemokine biomarkers derived from both the Control medium and TCM obtained from each individual cell line. This was accomplished using the Bead-Based Multiplex Assay (MILLIPLEX MAP Human Cytokine/Chemokine Magnetic Bead Panel - Premixed 41 Plex, cat. # HCYTMAG-60K-PX41, Millipore, Merck Russia) and Luminex technology with the QuattroPlex biomarker analysis system (QuattroPlexLab_comp, DIA-M, Russia). This innovative immunoassay uses microsphere technology with Luminex™ beads coated with specific antibodies to effectively capture analytes. A total of 25 μl from each secretome was utilized to identify the secreted factors. Human cytokine standards added to the antibody-conjugated beads were used to calibrate the system according to the standard protocols provided by the vendor. This study examined the differences and similarities among various media cytokine/chemokine constituents and their effects on vascularization and tumor cell behavior in CAM model.

**8.** **Vascular leakage assay (FITC-dextran permeability assay)**

To evaluate vascular permeability, a 70 kDa FITC–dextran solution (Sigma-Aldrich) was intravenously injected into the CAM vasculature on day 8 after tumor cell implantation (EMD16). Following incubation at 37 °C for 30 minutes, embryos were frozen and processed according to previously published protocols [5]. Briefly, glass capillary tubes (outer diameter 1.0 mm, inner diameter 0.6 mm) were manually trimmed and connected to a 30G insulin syringe using flexible silicone tubing to facilitate controlled injection. A 70 kDa FITC-dextran solution (Sigma-Aldrich) was prepared at a concentration of 25 mg/mL, and 100 μL was injected into each embryo. Following injection, embryos were to allow adequate systemic circulation of the dextran tracer. Subsequently, cryosectioning of tumor nodules were performed. Cryosections were stained with DAPI (1 μg/mL, 5 minutes), rinsed with PBS, and mounted for fluorescence imaging. Fluorescence images were acquired using a EVOS™ M5000 Imager microscope under FITC (green) and DAPI (blue) channels. Image analysis was performed using ImageJ software to quantify the total area of FITC fluorescence within the tumor region as a measure of vascular leakage. All imaging parameters were kept consistent across experimental groups.

**9.****Neutralization of VEGF in TCM**

To neutralize VEGFA in tumor-conditioned media (TCM), a clinically approved monoclonal antibody, bevacizumab (Avastin®, 25 mg/ml, 100 mg/4ml, concentrate for i.v. injection, Roche-Moscow, Russia), was added to both the experimental and rescue group TCMs at a final concentration of 20 ng/ml [6, 7], following filtration as described in Section 2.2. The CAM membranes were then pre-treated as outlined in Section 2.1. To validate the specific targeting of VEGFA, a rescue experiment was performed. U2OS cells were transfected with the FC-3398-VEGFA plasmid using Lipofectamine™ 3000 (Thermo Fisher Scientific, USA), allowing transient overexpression of human VEGFA driven by the CMV promoter. The FC-3398-VEGFA plasmid encodes human VEGFA, and was used to restore VEGFA expression in U2OS cells, a human osteosarcoma cell line, to maintain species consistency and experimental comparability. At 24 hours post-transfection, the cells were processed as described above to collect VEGFA-enriched conditioned media.

# Supplementary Figure 1


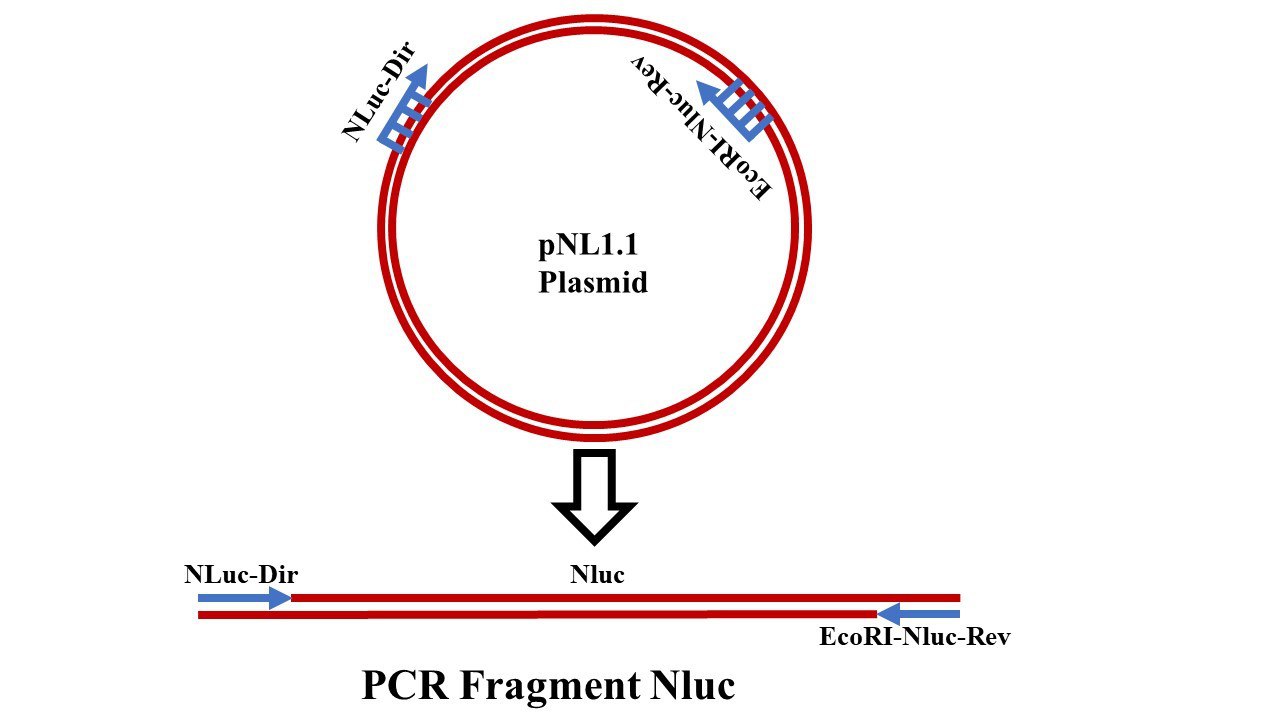


**Figure 1A. Schematic representation of the plasmid pNL1.1 and PCR amplification of the NanoLuc ORF.** The upper panel shows the plasmid map of pNL1.1, which encodes the NanoLuc luciferase gene and was used as the PCR template. Primer binding sites for amplification (Nluc-Dir and EcoRI-Nluc-Rev) are indicated. The lower panel illustrates the resulting PCR product containing the full-length NanoLuc open reading frame (ORF), flanked by the Nluc-Dir and EcoRI-Nluc-Rev primer sequences. This fragment was used for subsequent fusion with a T2A linker and subcloning into expression constructs.


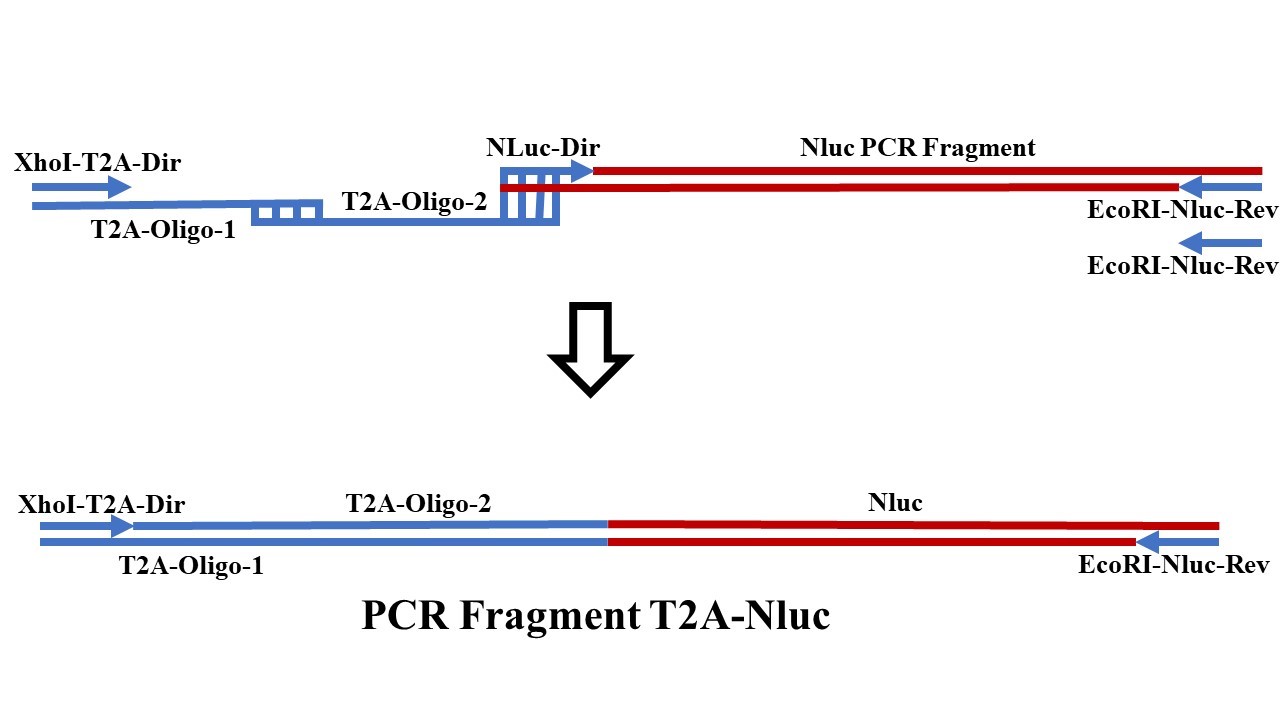


**Figure 1B. Schematic assembly of the T2A-Nluc PCR fragment.** The upper panel shows the strategy for constructing the T2A-Nluc PCR product by fusing a synthetic T2A sequence to the N-terminus of the NanoLuc open reading frame (ORF). The T2A region was generated by annealing two complementary oligonucleotides, T2A-Oligo-1 and T2A-Oligo-2, and combined with the NanoLuc PCR fragment using overlap extension PCR. The final amplification was performed using primers XhoI-T2A-Dir and EcoRI-Nluc-Rev. The lower panel represents the resulting full-length T2A-Nluc PCR product, flanked by XhoI and EcoRI restriction sites, used for subsequent cloning into the expression vector. This fragment encodes a self-cleaving 2A peptide linker followed by NanoLuc luciferase.

# Supplementary Figure 2


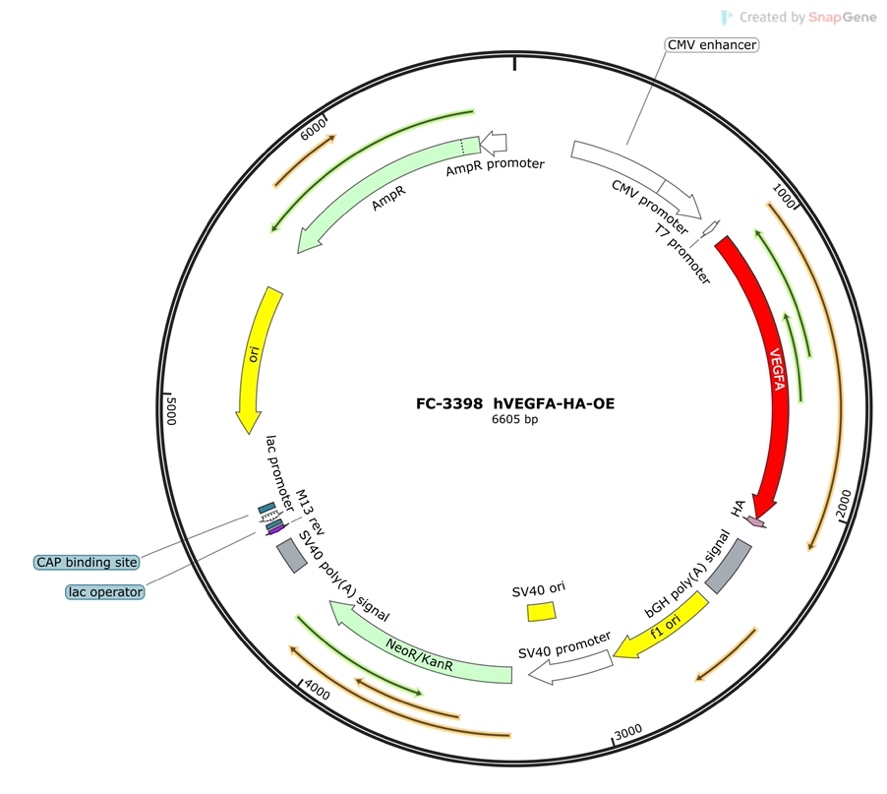


**Figure 2. Plasmid map of FC-3398 hVEGFA-HA-OE.** Schematic representation of the mammalian expression plasmid FC-3398 hVEGFA-HA-OE (6,605 bp), obtained from Fubio Biotechnology Co., Ltd. (Suzhou, China). This plasmid encodes human VEGFA tagged with an HA epitope under the control of a CMV promoter. Additional features include a bGH polyadenylation signal for mRNA stability, a Neomycin resistance gene (NeoR/KanR) for mammalian selection, and an SV40 origin of replication. The plasmid was used for transient overexpression of VEGFA in functional experiments.

# Supplementary Figure 3

In ovo bioimaging of tumor nodules formed at EMD 16

To validate whether fluorescence intensity on the CAM surface could be used for preliminary quantification of tumor nodules, the eggs implanted with 1.5x10^6^ WEHI 164-Kat2S-T2A-Nluc cells, along with a blank control group, were collected for fluorescence imaging. The fluorescent regions within the ring were delineated using the ROI (the green area) tool, and the integrated intensity (Sum intensity) within the ROI was calculated using an algorithm provided by the Icy software.

**
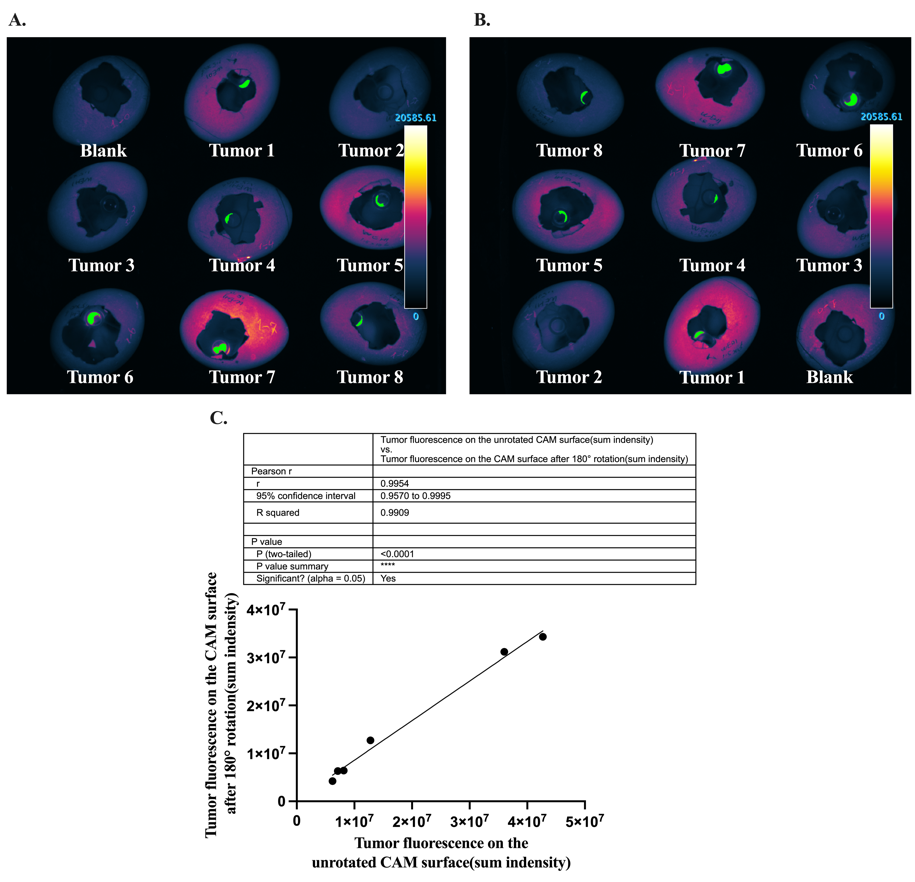
**

**Figure 3.** **In ovo fluorescence bioimaging and quantitative validation of tumor nodules on the CAM surface.** *In ovo* bioimaging of tumor nodules formed at EMD 16 after implantation of 1.5 x10^6^ WEHI 164-Kat2S-T2A-Nluc cells were obtained using LumoTrace® Fluo (Abisense LLC, Russia) imaging system. (A.) Eggs positioned at 0° rotation: the upper left corner – control egg, the rest (from left to right in each line) includes eggs of the experimental group 1 to 8; (B) eggs positioned at 180° rotation: the lower right corner – control egg, the rest (from right to left in each line) includes eggs of the same experimental group 1 to 8. Green regions indicate tumor ROI. (C) Correlation of tumor fluorescence on the unrotated CAM surface versus after 180° rotation. Each point represents a tumor formed by WEHI 164-Kat2S-T2A-Nluc cells. A strong linear correlation (r = 0.9964, P < 0.0001) confirms the consistency of fluorescence measurements under rotation.

To study how the irradiation angle affects the fluorescence of tumor nodules, the eggs were placed at various angles to the excitation light source. Figure S3 shows that after rotating the eggs by 180°, the ROI showed minimal change. This indicates that the fluorescence in the ring is related to the tumor nodules, not reflections.

# Supplementary Figure 4

To assess whether minimal mechanical injury combined with serum-free medium treatment could induce angiogenesis in the CAM microenvironment, an additional control experiment was performed(Figure S4). CAMs subjected to minimal scratching followed by treatment with serum-free unconditioned medium were compared with untreated CAMs over a 72-hour period. Quantitative analysis revealed no significant difference in vascularization between the two groups, indicating that neither minimal scratching nor serum-free medium exposure alone is sufficient to promote micro-vessel formation in the CAM.


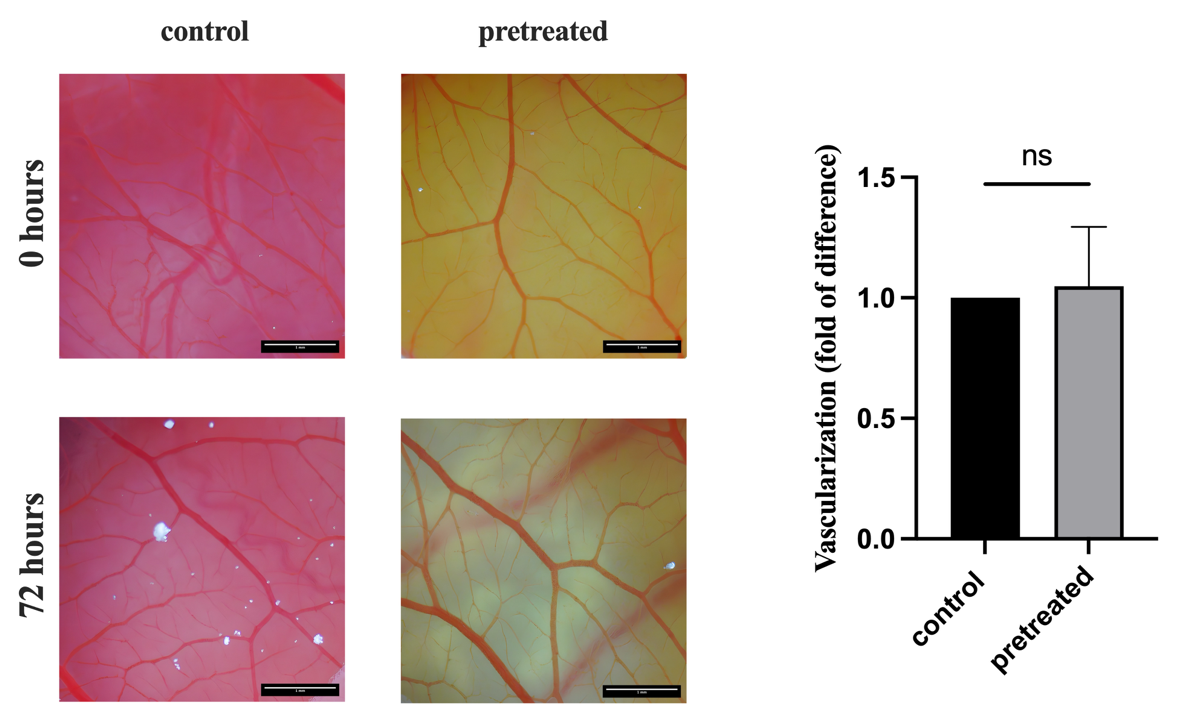


**Figure 4. Assessment of the effect of minimal scratching and serum-free medium treatment on CAM vascularization.** Representative CAM images acquired at 0 and 72 hours following minimal scratching combined with serum-free unconditioned medium treatment (pretreatment group) or no treatment (control group). Vascularization was quantified as fold change relative to the 0-hour time point. Comparison between the two groups revealed no statistically significant difference in micro-vessel density at 72 hours (ns). Scale bars = 1mm.

# Supplementary Table 1.

**Table 1**. Oligonucleotides, which are used in this study.

| **Oligonucleotide name** | **Oligonucleotide sequence** |
| --- | --- |
| Nluc-Dir | ATGGTCTTCACACTCGAAGAT |
| EcoRI-Nluc-Rev | ACTGCAGAATTCGATATCTTACGCCAGAATGCGTTCGCAC |
| XhoI-T2A-Dir | AATATACTCGAGCTGGCTCCG |
| T2A-Oligo-1 | AATATACTCGAGCTGGCTCCGGCGAGGGCAGGGGAAGTCTTCTAACATGCGGGGA |
| T2A-Oligo-2 | ATCTTCGAGTGTGAAGACCATTGGGCCGGGATTTTCCTCCACGTCCCCGCATGTTAGAAGACT |
| Kat-T2A-Nluc-Dir | ACTCATGGATCCACCGGTCGCCACCATGGTGG |
| Kat-T2A-Nluc-Rev | ACTGCAGAATTCGATATCTTACGC |

# Supplementary Table 2.

**Table 2**. Antibodies, which are used in this study.

| **Antibody** | **Fluorochrome** | **Brand** | **Catalog Number** | **Clone** |
| --- | --- | --- | --- | --- |
| Ki-67(Rat monoclonal) | — | eBioscience™,ThermoFisher scientific | 14-5698-82 | SolA15 |
| Anti-VEGFA (Rabbit mAb) | — | ServiceBio Inc. | GB15165 | — |
| Goat anti-Rat IgG H&L | Alexa Fluor® 647 | Invitrogen,ThermoFisher scientific | A-21247 | — |
| Goat anti-Rat IgG H&L | Alexa Fluor® 488 | Invitrogen,ThermoFisher scientific | A-11006 | — |
| Goat anti-Rabbit IgG H&L | Alexa Fluor® 647 | Invitrogen,ThermoFisher scientific | A-21245 | — |

# References

[1] B. Yuan, K. Shi, J. Zha, Y. Cai, Y. Gu, K. Huang, W. Yue, Q. Zhai, N. Ding, W. Ren, Nuclear receptor modulators inhibit osteosarcoma cell proliferation and tumour growth by regulating the mTOR signaling pathway, Cell Death & Disease, 14 (2023) 51.<https://doi.org/10.1038/s41419-022-05545-7>

[2] R. Al-Obaidy, A.J. Haider, S. Al-Musawi, N. Arsad, Targeted delivery of paclitaxel drug using polymer-coated magnetic nanoparticles for fibrosarcoma therapy: in vitro and in vivo studies, Scientific reports, 13 (2023) 3180.<https://doi.org/10.1038/s41598-023-30221-x>

[3] P. Nowak-Sliwinska, T. Segura, M.L. Iruela-Arispe, The chicken chorioallantoic membrane model in biology, medicine and bioengineering, Angiogenesis, 17 (2014) 779-804.<https://doi.org/10.1007/s10456-014-9440-7>

[4] T.D. Palmer, J. Lewis, A. Zijlstra, Quantitative analysis of cancer metastasis using an avian embryo model, JoVE (Journal of Visualized Experiments), (2011) e2815.<https://doi.org/10.3791/2815>

[5] W. Qin, W. Xu, L. Wang, D. Ren, Y. Cheng, W. Song, T. Jiang, L. Ma, C. Zhang, Bacteria‐elicited specific thrombosis utilizing acid‐induced cytolysin A expression to enable potent tumor therapy, Advanced Science, 9 (2022) 2105086.<https://doi.org/10.1002/advs.202105086>

[6] Ş. Comşa, R. Popescu, Ş. Avram, R.A. Ceaușu, A.M. Cimpean, M. Raica, Bevacizumab modulation of the interaction between the MCF-7 cell line and the chick embryo chorioallantoic membrane, in vivo, 31 (2017) 199-203.<https://doi.org/10.21873/invivo.11045>

[7] D. Shinde, Chicken embryo chorioallantoic membrane assay for pre-clinical evaluation of efficacy and safety of anti-angiogenic and hypoxic cell-starving tumor interventions, University of Zurich, 2014,
